# The Impact of the Swedish Care Coordination Act on Hospital Readmission and Length-of-Stay among Multi-Morbid Elderly Patients: A Controlled Interrupted Time Series Analysis

**DOI:** 10.5334/ijic.6510

**Published:** 2023-05-23

**Authors:** Douglas Spangler, Wilhelm Linder, Ulrika Winblad

**Affiliations:** 1 Department of Public Health and Caring Sciences, Uppsala University, Uppsala, Sweden

**Keywords:** care coordination, care integration, health policy, readmission, length of stay, multi-morbid elderly patients

## Abstract

Coordinating follow-up care after discharge from hospital is critical to ensuring good outcomes for patients, but is difficult when multiple care providers are involved. In 2018, Sweden adopted the Care Coordination Act, which modified economic incentives to reduce discharge delays and mandated a discharge planning process for patients requiring post-discharge social- or primary care services. This study evaluates the impact of this reform on hospital length-of-stay and unplanned readmissions among multi-morbid elderly patients.

Interrupted time series analysis of all in-patient care episodes involving multi-morbid elderly patients in Sweden from 2015 – 2019 (n = 2 386 039) was performed. Secondary analyses using case-mix adjustment and controlled interrupted time series analysis were employed to assess for bias.

Average length of stay decreased during the post-reform period, corresponding to 248 521 saved care days. Unplanned readmissions meanwhile increased, corresponding to 7 572 excess unplanned readmissions. While reductions in length-of-stay were concentrated among patients targeted by the reform, increases in readmission rates were similar in patients not targeted by the reform, indicating potential confounding. The reform thus appears to have achieved its goal of decreasing in-patient length of stay, but a robust effect on readmissions, outpatient visits, or mortality was not found. This may be due to lackluster implementation or an ineffective mandated intervention.

## Background

In response to aging populations and increasingly complex healthcare needs, many health- and social care systems are in the process of reconfiguring services to better serve patients with poor functional status, chronic disease, and multi-morbidity [1]. As these patients often require continuous services from both health- and social care, many healthcare systems struggle to maintain a high level of care quality for this patient population [2,3]. Adapting health- and social care to elderly, multi-morbid patients is thus an urgent issue for governments and healthcare providers.

Care integration between health- and social care has come to the forefront as a way to improve care for these particularly vulnerable patients. Poor coordination between health- and social care providers can be a contributing factor to prolonged hospital stays and readmission [4,5,6]. Elderly patients in particular are increasingly readmitted due to exacerbations of chronic ambulatory care sensitive conditions (ACSC) or acute conditions related to inadequate social support (e.g. falls, malnutrition, and dehydration) [7]. As such, improving the integration of hospital-, primary-, and social care has the potential to reduce hospital length of stay and readmissions [8]. Targeting potentially avoidable readmissions aligns the goals of both individual patients and health care systems as acute disease is prevented and expensive hospital services can be avoided [9].

Taken together, increasing the degree of care integration in healthcare systems offers a promising path to improving patient outcomes and quality of life, while simultaneously increasing the efficiency of health- and social care systems [10,11,12]. Numerous models of integrated care and collaboration exist, typically developed and implemented in specific contexts/settings with unclear potential for transferability [13]. Little consensus thus exists in regards to optimal models of integration, or even how to measure the concept [14,15].

## Models of integrated care

A key component of integrated care is collaboration between care providers. Through collaboration, synergic effects not otherwise attainable by individual organizations may be achieved, resulting in more effective care provision [16]. Collaboration can however be difficult to achieve in practice due to institutional, contextual, and professional barriers [17]. Particular institutional settings including decentralization and marketization of the public sector can contribute to the complexity of health and social care systems, further complicating inter-organizational collaboration.

A number of models have been proposed to identify successful approaches to improving care integration [1]. Common interventions to improve collaboration and integration of care at the patient level are the use of case managers, multi-disciplinary teams, geriatric risk assessments, and care planning meetings [18,19,20]. At the organizational level, integration can be achieved through financial incentive networks (e.g. Accountable Care Organizations), partnerships, and mergers [21].

While care integration is most commonly investigated at the patient and organizational level, it can also be pursued at the policy level. In Denmark, a structural reform in 2007 introduced municipal co-financing of patients cared for in regional hospitals in an effort to incentivize municipalities to increase disease prevention measures [22]. Despite an increase in efforts related to disease prevention in the municipalities, one study found no strong correlation between such measures and hospital admissions [23].

A Norwegian coordination reform in 2012 also incentivized collaboration between health- and social care [24]. As in the Danish reform, municipalities were mandated to co-finance hospital admissions to state-owned hospitals, and were financially penalized for so-called “discharge ready days” among patients who remained hospitalized while awaiting follow-up from primary- and social care. The Norwegian coordination reform also called for the establishment of a new intermediate care level including in-patient primary care wards [25]. Some studies of the Norwegian Coordination Reform indicate that it was associated with a decrease in hospital admissions, especially in elderly and patients with ACSCs [26,27], while another found that the reform was not associated with increases in readmissions or mortality [28]. While numerous clinic-level integrated care interventions have been found to improve patient outcomes, the ability of policy instruments at the national level to impact patient outcomes is thus unclear.

Further afield, care integration initiatives based on incentivizing provider activities are popular. In Germany for instance, a “shared health gain” reimbursement model combined with care management programs sought to improve both population health and health system cost efficiency [29]. In the United Kingdom, studies of integrated care have often reported interventions to be multifactorial, with typical models containing four to six elements [1]. A well-studied example of a financial incentive intended to improve care integration is the Hospital Readmission Reduction Program in the United States, which implemented fines based on adjusted hospital 30-day readmission rates [30]. While hailed for its success in reducing readmission rates [31], concerns have been raised over apparently corresponding increases in mortality rates for which hospitals are not penalized [32].

## Care integration in Sweden

Sweden has a universal, yet highly decentralized model of health- and social care governance. The responsibility for providing health care is primarily devolved to the 21 regions, while social- and eldercare is the primary responsibility of the 291 local municipalities. Regions and municipalities thus share the responsibility of providing care services, but differ in the level of health care provided as physicians are exclusively employed by regions whereas municipal health care is mainly provided by nurses (e.g. home based health care). Social care services provided by municipalities come mainly in the form of supported accommodations (e.g. nursing-, and short-stay homes), home care services, and equipment provision (e.g. home adaptation and personal safety alarms) [33].

As the funding and provision of health- and social care are the responsibility of regions and municipalities, the Swedish national government has a limited mandate to govern their organization and goals. Governance at the national level is therefore mainly effected through legislation, agreements, and targeted financial contributions [34]. Inter-organizational collaboration is thus primarily effected directly between regions and their constituent municipalities. Inadequate inter-organizational collaboration between regions and municipalities for the multi-morbid elderly is a longstanding issue in Sweden, and during the last three decades there have been several initiatives by the national government to improve the situation. Starting in 1990, municipalities were made liable for reimbursing regions for hospitalized patients that remained in the hospital five days after being declared discharge ready. In 2010, further legislation imposed stricter requirements for discharge care planning and coordination between regions and municipalities though modifications of the Social Services Act (1980:620) and Health and Medical Services Act (1982:763). Despite the efforts of the Swedish national government, a recent report by the commission investigating the Swedish response to the Covid-19 pandemic highlighted that integration of regional health- and municipal social care in Sweden remains poor [35].

### The Swedish Care Coordination Act

On January 1st, 2018, a new law seeking to address these issues went into effect. The Care Coordination Act (CCA) redefined the hospital discharge process for patients determined to require post-discharge services [36]. The explicit goals of the CCA were to improve the quality of care for patients requiring post-discharge services from health and/or social care providers, and to ensure that patients requiring such services were discharged as soon as possible from the hospital (2017:612 §2). The CCA sought to achieve these goals through two primary policy instruments:

#### Stricter financial penalties for hospital discharge delays

The CCA reduced the grace period for discharge ready patients from five to three days and made notification requirements stricter. This policy instrument thus sought to achieve a reduction in hospital length of stay by minimizing discharge delays. Through immediate notification of primary- and social care providers regarding admitted patients in need of support after discharge, activities related to transitioning the patient from hospital to in-home- or nursing home care was to be initiated at an early stage of the patients’ hospital episode. This policy instrument thus includes both financial penalties in the form of municipal co-payment for discharge ready patients, and measures to improve the preconditions for expeditious discharge planning.

#### A standardized process for discharge care planning

The CCA mandated a standardized process for collaboration between regions and municipalities during and after hospitalization. In brief, the CCA defined regional primary care provision organizations as being responsible for inviting the patient and all relevant care providers to a care planning conference and developing a “Coordinated Individual Plan” (CIP) for patients in need of post-discharge support from regional and municipal health- and social care. Discharge planning was previously the responsibility of the discharging hospital and municipal social services, mostly in the absence of representatives from the regional primary care system [37]. The standardized collaborative care pathway introduced by the CCA entailed that the primary care system was to assume responsibility for the patient during the discharge process. Together with the patient and social services, they were expected to establish patient goals and determine responsibilities for the patients continuing care, thereby ensuring adequate follow-up and improving post-discharge care quality.

Regions and municipalities were largely expected to implement the law within their current budgets and, if needed, by transferring funds from secondary care to primary care [38]. While individual regions have to varying extents implemented reimbursement schemes to encourage care providers to perform care planning interventions, the second policy instrument can be seen as an “unfunded mandate” in that the national government dictated a specific care process to be performed by care providers for which no specific funding was granted. Furthermore, while the CCA is legally binding, it does not enumerate explicit penalties for non-compliance. Reports on the implementation of the second policy instrument have suggested that coordination between care providers using CIPs was sporadic, and that routines and guidelines on the practical use of CIPs are uncommon [39,40]. In a recent evaluation, it was found that only 30% of patients had any written plan following discharge, also suggesting a relatively low level of uptake of the interventions mandated by the reform [41].

The impact of the CCA on patient outcomes has not yet been investigated in the scientific literature, and the evidence regarding the ability of this type of policy instrument to improve outcomes is weak. It is therefore important to evaluate the effects of such policy instruments formally to establish whether they are able to improve collaboration between health- and social care, and by extension improve measures of outcome and system efficiency.

## Aim

To determine the impact of the CCA on in-patient length of stay and unplanned hospital readmission rates among elderly multi-morbid patients.

## Methods

### Study design

An Interrupted Time Series (ITS) analysis of all multi-morbid elderly patients discharged from in-patient care between 2015 and 2019 in Sweden. This analysis was complemented by case-mix adjusted models and a Controlled Interrupted Time Series (CITS) analysis to assess for confounding by simultaneous interventions or population changes (history bias) [42,43,44].

Case mix-adjustment was performed by including potential confounding or mediating factors as covariates in the base ITS models. Previous research has found that not only health-related factors, but also a patient’s socio-economic situation can impact the outcomes studied, such as readmissions and length of stay [45,46]. Case-mix adjusted models were thus developed to account for patient age, gender, marital status, type of admission (planned or unplanned), number of diagnoses/hospital interventions, the presence of an ACSC per the definition in Purdy et al. [47], and the type of social services used by the patient at the time of the care episode. We sought to control for country of birth, but could not due to a high level of missing data in 2015 (28%, see Appendix 2).

As described above, both major policy instruments of the CCA target patients which require cooperation between regional (i.e. hospital or primary) care, and municipal health and social care services. The full ITS study sample was therefore divided into two groups which were expected to be differentially impacted by the reform: Those with municipal health- and/or social care services at the time of discharge and were thus the primary target of the reform, and those without. The difference in the effect of the reform between these groups was then assessed using a Controlled Interrupted Time Series analysis. In this analysis we thus assume that patients registered to receive municipal social care services during the month of their discharge from hospital were more likely to be impacted by the changed financial penalties and to receive a CIP (hereafter referred to as the *“target group”*). More precisely, social services included being a resident of a nursing home, receiving municipal home care or social assistance services, or having a safety alert system at the time of discharge. Receiving a CIP, which includes collaboration between healthcare and social care providers, is expected to decrease readmissions by improving the provision and timeliness of services. Patients not registered to receive any municipal social- or health care services, hereafter referred to as the *“non-target”* group, are assumed to be less likely to be impacted by the financial penalties or receive a CIP while the same time being exposed to the same confounding events (e.g. changes in the number of available hospital beds during the study period) [42].

A protocol describing the study design and hypotheses was pre-registered [48], and ethics approval for the study was granted by the Swedish national ethics review authority (dnr 2019-04191).

### Study sample

Using inpatient admissions as the unit of analysis, the study sample included all hospital admissions for patients at least 65 years of age and with 2 or more distinct ICD-10 diagnosis codes documented during the care episode. The data used in this study were extracted from national registries managed by the National Board of Health and Welfare (NBHW), which are updated yearly based on data provided by each of the 21 Swedish regions. Data from the national in-patient care registry formed the main dataset for the study, and all in-patient care episodes for individuals meeting the inclusion criteria between January 1, 2015 and December 31, 2019 were extracted. The NBHW cause of death registry and outpatient care registry were linked to this dataset to provide data on 30-day mortality and outpatient care contacts respectively. Further data were extracted from the NBHW Social services and municipal health services registries to identify patients with social/municipal care needs at the time of discharge. Researchers with appropriate ethics approval may obtain the dataset from the NBHW with reference to “Dnr 5102/2020”, and reproduce the findings reported here using the R code provided as appendix 1. Aggregate monthly data is also provided in appendix 2.

A number of exclusion criteria were then applied to this dataset. Admissions were excluded based on having a missing variable necessary to perform data linkage or adjustment for clustering (hospital or hospital wards), and admissions ending in mortality. Upon receiving data from the NBHW, it was found that admissions towards the end of the study time-frame were substantially shorter on average owing to an artefact of how the NBHW processes data in yearly batches. This entailed that only admissions where the patient was discharged prior to the next year were included in the dataset, while patients with longer stays were not, thus inducing a bias in the dataset with regards to length of stay. To ensure that this source of bias did not impact the results, stays exceeding a set number of days and a corresponding interval of time at the end of the study were excluded. Based on exploratory analysis, an interval of 90 days was selected to minimize exclusions due to length of stay.

### Outcome measures

The goal of the first policy instrument was to reduce the amount of time spent in hospital after being formally denoted as ready for discharge. This measure is publicly reported at the aggregate level, and a clear drop following the reform may be seen [49]. However, the extent to which this translates to an actual reduction in inpatient length of stay is unclear. The total length of stay was thus selected to measure the impact of the first policy instrument.

Operationalizing an outcome measure for the second policy instrument is less straightforward. We hypothesized that if successful, this policy instrument was likely to reduce potentially avoidable readmissions, as gaps in medical follow-up after discharge are thought to be a major contributing factor to readmission rates [9,50]. Specifically, 30-day unplanned readmissions were selected as the primary outcome measure due to its prevalence in the literature on follow-up care quality [51,52]. Secondary outcomes were selected to provide a more comprehensive picture of the reform’s effect, and to assess for potential competing outcomes including 30-day mortality rates, as well as planned and unplanned (typically Emergency Department) outpatient care contacts.

### Statistical analyses

Descriptive statistics were generated and presented graphically using 28-day (4 week) rolling averages in the manuscript, and numerically in appendix 1. Analytical results were presented graphically by plotting point estimates of model fixed effects, as well as numerically using coefficient tables reporting the confidence intervals from which we draw our conclusions.

In the ITS analysis, we considered both level and trend changes to be plausible, given that immediate level-changes were apparent in public data regarding length of stay, while institutional inertia and a staggered roll-out of interventions implementing the CCA was likely to manifest as trends at the national level. The interruption was set to January 1, 2018, the date when the reform went into effect, and coefficients were defined to capture the pre-reform trend, level change at the time of the reform, and change in trend during the post-reform period.

The base ITS models and case mix-adjusted models were defined as additive, while the fixed portion of the CITS models included interaction effects between the level and slope terms and membership in the target group. Prior to estimating the analytical models, clustering at the regional, municipal, hospital, hospital ward, and individual levels was assessed. Clustering at the hospital ward level was found to be the most substantial source of dependency between observations and was therefore accounted for along with seasonal effects in all models. Models including random effects at the individual level (accounting for repeat visits by the same individual) were also estimated and are reported in appendix 1. Due to low levels of missing data, listwise deletion was used instead of multiple imputation.

We considered fixed effect coefficients excluding the null with 95% confidence (corresponding to a p-value of 0.05) to be statistically significant. Heirarchal linear regression was used estimate models, with percentile confidence intervals based on 1001 non-parametric bootstrap replicates of each model to account for skewness in model residuals. The statistical analysis followed the general approach to multilevel modelling described by Gelman and Hill [53]. All data processing and analyses were performed in R (v. 4.2.2) using the *lme4* package [54] to estimate models and the *lmeresampler* package [55] to generate cluster bootstrapped confidence intervals.

## Results

### Descriptive statistics

**Figure 1:**
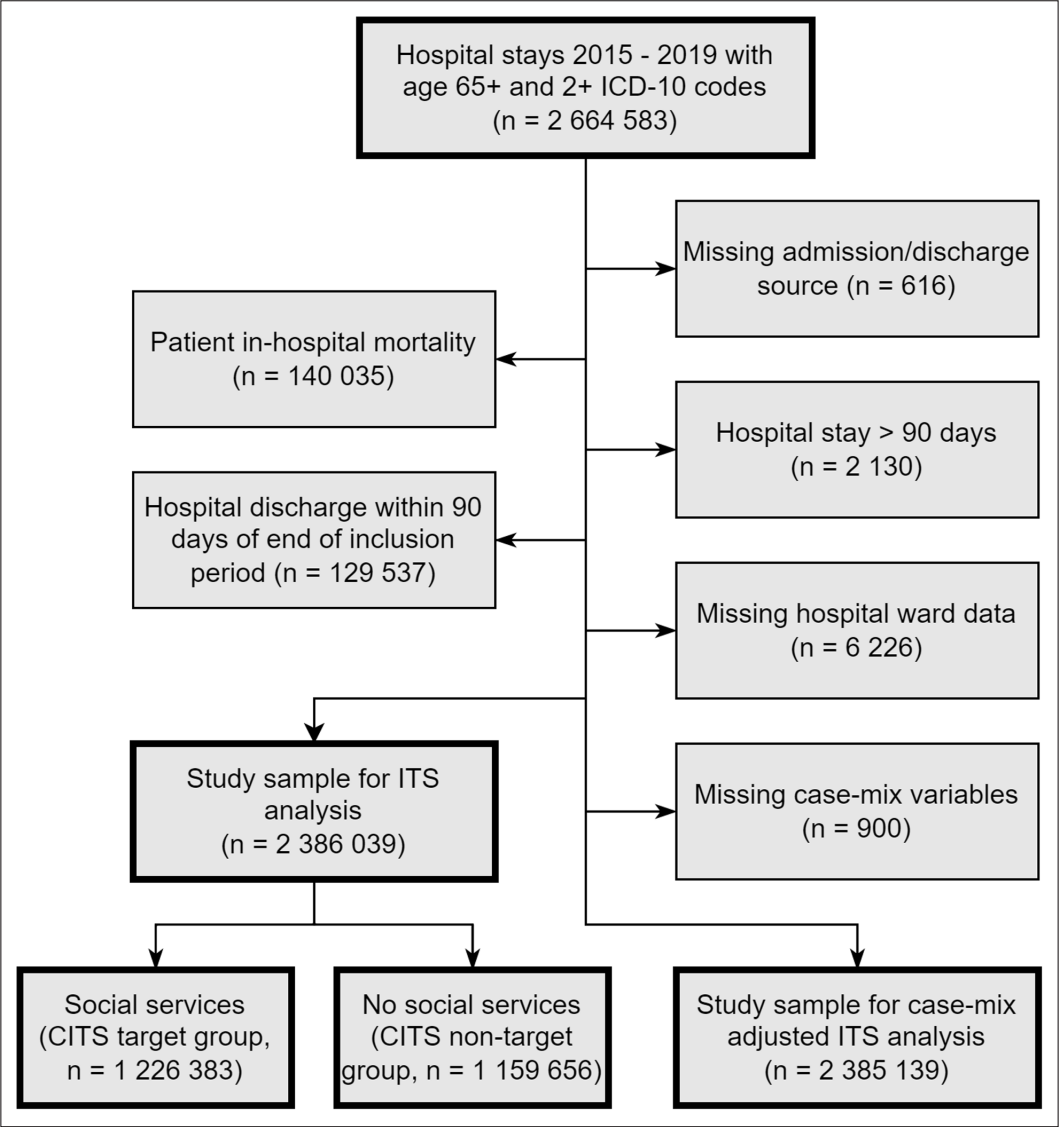
Inclusion/exclusion criteria flow chart.

Per Figure 1 below, a total of 2 664 583 hospital records between 2015 and 2019 met study inclusion criteria. 616 records were excluded due to missing admission/discharge source or hospital. 140 035 records were excluded due to the death of the patient prior to discharge, corresponding to an in-hospital mortality rate of 5.25%. 2 130 hospital stays over 90 days were excluded, as well as 129 537 records from the final 90 days of the dataset. Finally, 6 226 observations with no documented hospital ward were excluded, resulting in a final sample size of 2 386 039. Of these, 900 observations were missing a case-mix adjustment variable, and were excluded from the case-mix adjusted models.

**Figure 2:**
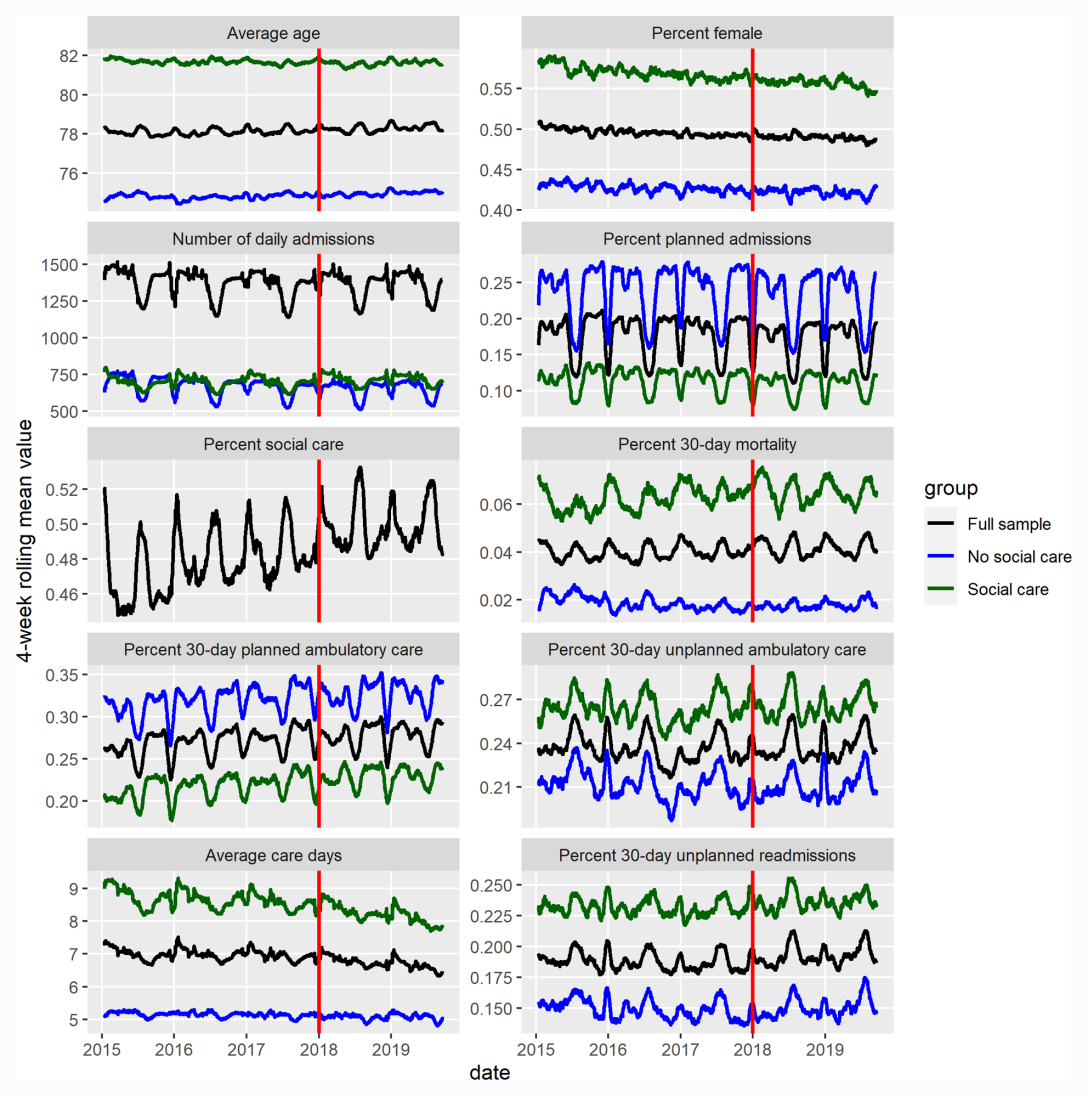
Descriptive statistics for the study sample used in the ITS-analysis and CITS-analysis (social care target group and no social care non-target group). All data presented using 28-day (4 week) rolling average values. Red vertical line denotes the time of intervention.

Study sample characteristics are descibed in Figure 2, where clear seasonal patterns may be discerned for all measures with the exception of patient sex. The number of included records tended to dip during the summer months of July and August, dropping to 37 658 during these two months from a baseline of 41 864, as well as over the winter holidays. The average age of the patients included in the study was 78.4 years, with a slight increasing trend and biannual seasonality, with the age of patients peaking during both the summer and winter months. Over the course of the study period, the patient cohort shifted from 51% females in January, 2015, to consisting of 48.8% females in September 2019. An average of 51.4% of included patients received municipal social services, and the measure tracked changes in patient age closely, displaying similar biannual seasonality and an upward trend. 30-day post-discharge mortality averaged 4.4%, following similar seasonal trends as age. Overall seasonality appeared to be strongly associated with summer and winter holidays, when fewer planned (lower risk) admissions occurred. The bottom two facets of Figure 2 present the primary outcomes of 30-day readmissions and length of stay which will be evaluated in the following sections. Readers seeking further descriptive analysis may refer to the aggregate panel data covering all variables employed in this analysis provided as appendix 2.

### Analytical results

**Figure 3:**
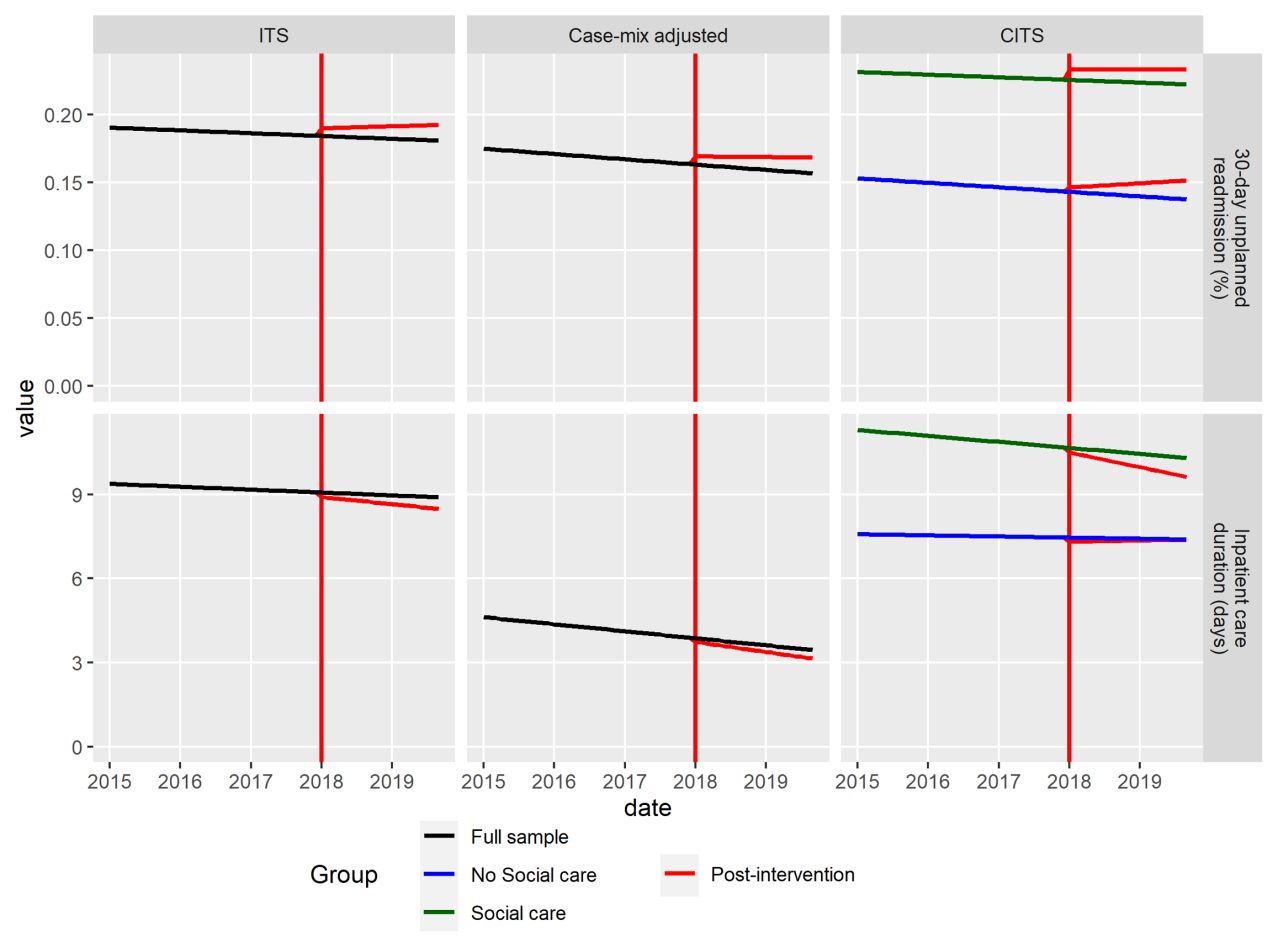
Results of the ITS-analysis (left), case-mix adjusted ITS-analysis (middle) and CITS-analysis (right) for 30 day unplanned readmission (top) and inpatient length of stay (bottom). Note that intercepts differ from the averages presented in figure 1 due to the use of varying random intercepts in all models, and to the estimation of effects at the mode value of covariates in the case-mix adjusted models.

The results of the statistical analysis are summarized in Figure 3 below. Briefly, the *ITS analysis* of all included admissions suggested that unplanned readmissions increased following the intervention (top row), while average inpatient length of stay decreased over time (bottom row). The *Case-mix adjusted* analysis largely reflected the results of the base analysis, though effects were somewhat diminished. The *CITS analysis* found that similar increases in readmission rates occurred in both target- and non-target groups, while reductions in length of stay were found only in the target group of the reform. In the following sections, the results are reviewed in more detail.

### ITS analysis

Table 1 presents model coefficients for the base analysis. The findings suggest that readmissions saw a level increase of 0.571 (95% CI 0.108 – 1.039) percentage points, corresponding to a 3% increase relative to the December 2017 readmission rate of 18.9%. No significant difference in post-intervention slope was identified. Length of stay saw a level-change of –0.156 (–0.259 – –0.055) days over the post-intervention period, and a divergence from the pre-intervention trend of –0.013 (–0.019 – –0.006) days per month.

**Table 1:** ITS analysis fixed effect coefficients in full study sample.


COEFFICIENT	30-DAY UNPLANNED READMISSION (PERCENT, 95% CI)	INPATIENT LENGTH OF STAY (DAYS, 95% CI)

Intercept	**19.047 (18.642 – 19.382)**	**9.381 (9.254 – 9.535)**

Change per month since start	**–0.017 (–0.03 – –0.004)**	**–0.009 (–0.013 – –0.005)**

Post-intervention level change	**0.571 (0.108 – 1.039)**	**–0.156 (–0.259 – –0.055)**

Post-intervention change per month	0.029 (–0.004 – 0.06)	**–0.013 (–0.019 – –0.006)**


Effects with 95% confidence intervals excluding zero are written in bold text. Coefficients represent average effects of the reform in the full study sample.

In cumulative terms, by the end of the study period 30-day readmissions were 1.11 percentage points (9%) higher than they would have been if the pre-intervention trend had continued, while the average in-patient care stay was 9.3 hours shorter. Within the sample this study investigates, this corresponds to 248 521 saved care days over the course of the 21-month post-intervention study period. However, these findings also suggest that an additional 7 572 readmissions within 30 days were generated, corresponding to an additional 58 586 care days, assuming that the length of stay of these additional readmissions were similar in length to the average post-intervention readmission (7.7 days).

### Case-mix adjusted ITS analysis

Table 2 presents the ITS analysis coefficients in the context of risk-adjusted models accounting for individual-level demographic and clinical characteristics. Results of the case-mix adjusted analysis were in line with the results of the unadjusted base models, though the magnitude of the intervention effect on length of stay was somewhat diminished.

**Table 2:** Case-mix adjusted ITS analysis fixed effect coefficients.


	COEFFICIENT	30-DAY UNPLANNED READMISSION (PERCENT, 95% CI)	INPATIENT LENGTH OF STAY (DAYS, 95% CI)

	Intercept	**20.879 (19.23 – 22.543)**	**5.445 (5.163 – 5.739)**

Study effects	Change per month since start	**–0.033 (–0.046 – –0.019)**	**–0.021 (–0.026 – –0.016)**

	Post-intervention level change	**0.593 (0.143 – 1.043)**	**–0.129 (–0.246 – –0.012)**

	Post-intervention change per month	0.028 (–0.006 – 0.058)	**–0.009 (–0.017 – –0.002)**

Covariates	Age	**–0.094 (–0.114 – –0.073)**	**–0.036 (–0.039 – –0.032)**

	Female	**–2.018 (–2.26 – –1.789)**	**0.094 (0.061 – 0.127)**

	Civil status: Married (ref)		

	Civil status: Divorced	0.181 (–0.13 – 0.486)	**–0.054 (–0.094 – –0.012)**

	Civil status: Unmarried	–0.059 (–0.457 – 0.302)	**0.145 (0.08 – 0.21)**

	Civil status: Widow	**–1.198 (–1.467 – –0.918)**	**–0.11 (–0.149 – –0.07)**

	Planned admission	**–5.281 (–5.581 – –4.923)**	**–1.617 (–1.721 – –1.501)**

	Number of diagnoses	**1.152 (1.101 – 1.207)**	**0.591 (0.572 – 0.611)**

	Number of Interventions	**–0.229 (–0.288 – –0.168)**	**1.344 (1.313 – 1.374)**

	Ambulatory Care Sensitive Condition	**0.629 (0.203 – 1.056)**	**–0.186 (–0.254 – –0.124)**

	Social services: None (ref)		

	Social services: In-home care	**8.759 (8.436 – 9.091)**	**2.207 (2.113 – 2.299)**

	Social services: Nursing home	**3.218 (2.702 – 3.76)**	**4.954 (4.733 – 5.191)**


Effects with 95% confidence intervals excluding zero are written in bold text. Study effect coefficients represent average effects of the reform in the full study sample.

While we report coefficients for covariates used in the case-mix adjustment for the sake of transparency, we urge caution in their interpretation. While the inclusion of each covariate is believed to be justified in terms of its causal relationship to the intervention and outcome (See the causal graph provided in appendix 1), the complex causal interactions between the covariates makes interpretation difficult. In terms of overall predictive value, these models differed substantially, with conditional pseudo r-squared values of 0.55 for length of stay and 0.046 for readmission rates.

### CITS analysis

In the CITS analysis, the impact of the reform on the portion of the ITS study sample which was targeted by the reform (hospital visits by patients with municipal social care services) were compared with those not targeted (those without municipal social care services). Substantial differences were found with regards to patients with and without municipal social services at the time of discharge as presented in Table 3 below.

**Table 3:** CITS analysis fixed effect coefficients.


	COEFFICIENT	30-DAY UNPLANNED READMISSION (PERCENT, 95% CI)	INPATIENT LENGTH OF STAY (DAYS, 95% CI)

Main effects in non-target (no social care) group	Intercept	**15.319 (14.896 – 15.743)**	**7.578 (7.459 – 7.726)**

	Change per month since start	**–0.028 (–0.044 – –0.011)**	–0.003 (–0.007 – 0)

	Post-intervention level change	0.322 (–0.248 – 0.884)	**–0.145 (–0.239 – –0.03)**

	Post-intervention change per month	**0.053 (0.013 – 0.089)**	0.007 (–0.001 – 0.012)

Coefficient interaction effect in target (social care) group	Intercept	**7.828 (7.27 – 8.294)**	**3.731 (3.489 – 3.913)**

	Change per month since start	0.011 (–0.015 – 0.037)	**–0.014 (–0.02 – –0.006)**

	Post-intervention level change	0.447 (–0.502 – 1.397)	–0.018 (–0.26 – 0.155)

	Post-intervention change per month	–0.036 (–0.094 – 0.017)	**–0.033 (–0.044 – –0.019)**


Effects with 95% confidence intervals excluding zero are written in bold text. Main effect coefficients represent average effects in the non-target population, and coefficient interactions may in this case be interpreted as the difference between non-target and target populations – The interaction effect point estimate added to the main effect point estimate equals the effect size in the target group.

Readmission rates for patients with social services at discharge were substantially higher than those for patients without the need for social services (7.828 (7.27 – 8.294) percentage points), and average length of stay for patients with social services was 3.731 (3.489 – 3.913) days longer than patients without social services at discharge. No significant differences were identified with regards to readmission rates between the target and non-target groups with regards to level or slope changes. Regarding length of stay, both groups experienced similar instantaneous decreases in length of stay of –0.145 (–0.239 – –0.03) days following the intervention. Only patients receiving social services however experienced a reduction in inpatient stays over time, estimated at –0.033 (–0.044 – –0.019) days per month. We also found that applying case-mix adjustment to the above CITS models did not affect the interpretation of our findings (see Appendix 1), which supports the findings of the ITS analysis with regards to length of stay being causally attributable to the CCA reform. The results of additional pre-specified secondary analyses which may be found in appendix 1 regarding the effects among patients with different levels of social care (in-home vs. nursing home) furthermore suggest stronger intervention effects among patients with more intensive forms of social care.

### Secondary analyses

In addition to the primary outcomes, a number of secondary outcomes were specified *a priori* to investigate potential effects of the reform. These included impacts of the reform on mortality, as well as planned and unplanned out-patient care visits.

We did not identify an overall impact of the reform on mortality rates. In the CITS analysis however, we found that patients with social care experienced greater reductions in 30-day mortality rates than those without. Effects found in CITS analyses without corresponding effects in the primary analysis are however typically considered to be of limited evidentiary value [42].

With regards to out-patient visits, we identified an increase in the rate of unplanned out-patient visits within 30-days of discharge and a reduced rate of planned out-patient visits, which ran counter to our a priori hypothesis that a successful implementation would increase the proportion of planned visits. Similar to our findings regarding unplanned readmission rates however, these results were not robust upon secondary CITS analysis. Given the lack of robust findings among these secondary outcomes, we do not emphasize them in this manuscript, but summaries of model estimates may be found in appendix 1.

In a sensitivity analysis investigating the impact of accounting for clustering effects at the individual level, we found that the statistical significance of the level change in 30-day readmissions in the unadjusted ITS model was reduced to the point that a null effect could not be excluded with 95% confidence. The remainder of the findings were unaffected.

## Discussion

### Key results

This study presents strong evidence that the CCA reduced the average hospital length-of-stay, with our model estimating a total savings of 248 521 care days over the 21-month post-intervention period in the ITS study sample. These findings were robust in both case mix-adjusted and CITS analyses. With regards to post-discharge outcomes however, the evidence is more mixed. The ITS and case mix-adjusted ITS results indicate an increased rate of unplanned readmissions within 30 days of hospital discharge. However, CITS analysis indicated that the effect was similar in patients targeted and not targeted by the policy instruments of the reform, indicating potential confounding.

#### Interpretation

The findings suggest that the instruments of the CCA regarding discharge-ready patients had a robust ability to reduce inpatient length of stay. The mechanism of sharpening penalties and shifting payment responsibility towards the care provision organizations responsible for arranging post-discharge care, i.e. the municipalities, from five to three days thus appears to have been effective.

The mandate to follow a standardized care planning process upon discharge (e.g. the introduction of the CIP) did not demonstrate robust impact in terms of improving post-discharge outcomes, such as lowered readmissions, reduced mortality, or a shift towards planned over unplanned outpatient care. Both the ITS and case-mix adjusted ITS analyses rather demonstrated increases in readmissions after the introduction of the CCA, and no change in post-intervention slope. However, the CITS-analysis found similar effect sizes in targeted and non-targeted populations. This suggests that the findings of the ITS analysis may have been confounded due to history bias, or that the assumption of differential effects in the target and non-target groups was fallacious.

Post-discharge care planning was previously the responsibility of the discharging hospital, but is in the framework of the CCA now the responsibility of the patient’s primary care provision organization, and it may be that these actors have not yet fully embraced this role. Unfortunately, high-quality data is not available at the national level regarding the actual use of CIPs upon hospital discharge. The findings are thus inconclusive as to whether the lack of impact of the reform is due to the mandated care planning process itself being ineffective, or the result of a lackluster implementation of the interventions mandated by the CCA. Further research is needed to investigate the extent to which the prescribed processes have actually been implemented in the regions.

While some funding was provided to coordinate efforts, the regions and municipalities mandated to implement the new care planning process were not provided with national funding to do so. This study thus casts doubt on the efficacy of so-called “unfunded mandates” by national-level actors upon lower-level health- and social care providers to implement specific care processes. The results suggest that aligning financial incentives with desired societal outcomes may be a more effective option in achieving reformatory ends on the part of central governments in decentralized healthcare systems. While such financial incentives are demonstrably effective at achieving their explicit quantitative goals [31], care must be taken that they do not result in poorer outcomes in non-incentivized measures of quality [56].

While we found no improvement in post-discharge outcomes following the reform, we also did not find robust evidence of harm. It is tempting to attribute an increase in readmissions found in this study to an increase in premature discharges of patients following the reform. If this was the case, we would have expected to see readmission increase gradually over time in concert with the reductions in length of stay, and for the increase in readmissions to be concentrated among patients with social care needs, i.e. the group whose length of stay decreased. Given that we did not find such results, this argument is not supported by the data. A possible explanation of the increased level of readmissions after the introduction of the CCA could be that previously utilized discharge routines may have been abandoned without adequate implementation of the CIPs.

As reflected in our results, findings from the Scandinavian countries are generally inconclusive regarding the effect of similar reforms on readmission rates. Unlike the Norwegian Coordination Reform [25], the CCA was not associated with a decrease in hospital readmissions. A key difference is that the Norwegian reform included the introduction of intermediate care facilities with inpatient capabilities run by primary care physicians. The scientific literature on reforms in these countries does however increasingly point to the effectiveness of utilizing financial incentives in policymaking to stimulate impact of new reforms [22,23,25].

## Strengths and Limitations

Susceptibility to history bias (i.e. simultaneous interventions or shifts in population characteristics) is the primary weakness of ITS analysis. A strength of the study is that multiple forms of pre-specified secondary analyses to investigate whether this source of bias influenced the primary results were performed. The CITS analysis for instance casts doubt on the causal link between the reform and the increase in 30-day readmissions.

A limitation of the study is that the CITS analysis is based on groups which differ in their theoretical likelihood of being impacted by the CCA, rather than the actual provision of the interventions prescribed by the CCA. While this approach is similar to the comparison groups used by Ambugo and Hagen to evaluate similar reforms in Norway [28], it nonetheless entails that the findings rest on a theoretical assumption rather than identification of actual treatment effects. Per the pre-registered study protocol [48], it was planned to include additional comparison groups including patients with and without care planning interventions (i.e. CIPs), and patients with and without ACSCs. Upon receipt of the data however, it was found that documentation of care planning meetings in hospital records was rare, with a total of only ca. 5 000 instances of documented care planning meetings among the 2.6 million analyzed records. We also found that ACSCs lacked differential intervention effects in any of our analyses, suggesting that the presence of an ACSC was not a good proxy measure for exposure to the intervention. While we investigated the use of machine learning models to increase the predictive power of the adjusted models per our analysis plan, we found that results were substantively similar to those obtained using simpler multivariable regression models. We thus opted for this simpler approach, and will investigate the use of more complex models to predict length of stay and readmission in future studies.

A further limitation of the study is that the post-intervention time frame is only 2 years long. It may be that benefits from the reform in terms of patient outcomes only become apparent over longer time-frames. However, due to the substantial confounding effects of the Covid-19 pandemic on this population, including additional data from 2020 and beyond is unlikely to be useful.

## Conclusion

This study suggests that a policy instrument involving financial incentives was successful in reducing inpatient length of stay. However, the study did not identify an improvement in patient outcomes such as readmission or mortality following the introduction of a standardized post-discharge care pathway, possibly due to lackluster implementation or an ineffective mandated intervention. These findings highlight the difficulty of implementing detailed national-level steering mechanisms in decentralized healthcare systems.

## Additional Files

The additional files for this article can be found as follows:

10.5334/ijic.6510.s1 Appendix 1. Analysis notebook.

10.5334/ijic.6510.s2 Appendix 2. Supplementary aggregate panel data.
