# Supplementary material for: The Impact of the Swedish Care Coordination Act on Hospital Readmission and Length-of-Stay among Multi-Morbid Elderly Patients: A Controlled Interrupted Time Series Analysis
Source: Int J Integr Care. 2023 May 23;23(2):17. doi: 10.5334/ijic.6510 (PMC10216000; doi:10.5334/ijic.6510)
Supplement: Appendix 1. — Analysis notebook. [file ijic-23-2-6510-s1.zip › AppendixFiles/Appendix.html]

Appendix - The impact of the Swedish Care Coordination Act on hospital readmission and length-of-stay: A controlled interrupted time series analysis


# Appendix - The impact of the Swedish Care Coordination Act on hospital readmission and length-of-stay: A controlled interrupted time series analysis

#### Redacted for blinding

- Setup
- Session information
- Load data
- Estimate models
- Calculate bootstrap CIs
- Tables and figures
  - Figure 2 - Aggregate
    data desription
  - Figure 3 - Model
    summaries
  - Table 1 - Base model
    coefficients
  - Table 2 - Risk
    adjusted model coefficients
  - Table 3 -
    Control group model coefficients
- Additional analyses
  - Causal graph
  - Population description
  - Results noted in text
  - Social care levels
  - Undifferentiated
    readmissions
  - Case-mix
    adjusted control group analysis
  - Secondary outcomes
  - Aggregate data analysis
  - Indivudual-level
    clustering

This document fully specifies the data processing steps and analyses
reported in the study noted above. The findings may thus be reproduced
by obtaining the original raw data from the Swedish National Board of
Health and Welfare with reference to dnr 5102/2020. A prespecified
secondary analysis based on aggregated data which can be shared publicly
is also provided.

# Setup

```
# Load packages (all available on CRAN and installable using install.packages())
library(tidyverse)
library(lubridate)
library(lme4)
library(lmeresampler)
library(foreach)
library(doParallel)
library(zoo)
library(MuMIn)
library(data.table)
library(multidplyr)
library(knitr)
library(readxl)
library(MASS)
library(lmtest)
library(sandwich)

# Set working directory to file location (only works if you're using Rstudio! Set this manually otherwise)
knitr::opts_knit$set(root.dir = rprojroot::find_rstudio_root_file())

# Don't use scientific notation
options(scipen = 999)

# Define functions for use later
elapsed_months <- function(end_date, start_date) {
  ed <- as.POSIXlt(end_date)
  sd <- as.POSIXlt(start_date)
  12 * (ed$year - sd$year) + (ed$mon - sd$mon)
}

boot_parallel <- function(mod,cl,n_sim = 143,n_core = 7,samp_struct = c(T,T,T)){ # 143*7 = 1001
  out <- foreach(
    B = rep(n_sim, n_core),
    .combine = combine_lmeresamp,
    .packages = c("lmeresampler", "lme4")
  ) %dopar% {
    bootstrap(mod, .f = fixef, type = "case", B = B, resample = samp_struct)
  }
  return(out)
}
```

# Session information

```
sessionInfo()
```

```
## R version 4.2.0 (2022-04-22 ucrt)
## Platform: x86_64-w64-mingw32/x64 (64-bit)
## Running under: Windows 10 x64 (build 19045)
## 
## Matrix products: default
## 
## locale:
## [1] LC_COLLATE=Swedish_Sweden.utf8  LC_CTYPE=Swedish_Sweden.utf8   
## [3] LC_MONETARY=Swedish_Sweden.utf8 LC_NUMERIC=C                   
## [5] LC_TIME=Swedish_Sweden.utf8    
## 
## attached base packages:
## [1] parallel  stats     graphics  grDevices utils     datasets  methods  
## [8] base     
## 
## other attached packages:
##  [1] sandwich_3.0-2     lmtest_0.9-40      MASS_7.3-56        readxl_1.4.0      
##  [5] knitr_1.39         multidplyr_0.1.1   data.table_1.14.2  MuMIn_1.47.1      
##  [9] zoo_1.8-11         doParallel_1.0.17  iterators_1.0.14   foreach_1.5.2     
## [13] lmeresampler_0.2.2 lme4_1.1-29        Matrix_1.4-1       lubridate_1.8.0   
## [17] forcats_0.5.1      stringr_1.4.0      dplyr_1.0.9        purrr_0.3.4       
## [21] readr_2.1.2        tidyr_1.2.0        tibble_3.1.7       ggplot2_3.3.6     
## [25] tidyverse_1.3.1   
## 
## loaded via a namespace (and not attached):
##  [1] nlme_3.1-157         fs_1.5.2             httr_1.4.3          
##  [4] rprojroot_2.0.3      tools_4.2.0          backports_1.4.1     
##  [7] bslib_0.3.1          utf8_1.2.2           R6_2.5.1            
## [10] DBI_1.1.2            mgcv_1.8-40          colorspace_2.0-3    
## [13] ggdist_3.2.0         withr_2.5.0          tidyselect_1.1.2    
## [16] compiler_4.2.0       cli_3.3.0            rvest_1.0.2         
## [19] xml2_1.3.3           HLMdiag_0.5.0        sass_0.4.1          
## [22] scales_1.2.0         digest_0.6.29        minqa_1.2.4         
## [25] rmarkdown_2.14       pkgconfig_2.0.3      htmltools_0.5.2     
## [28] dbplyr_2.2.0         fastmap_1.1.0        rlang_1.0.2         
## [31] rstudioapi_0.13      jquerylib_0.1.4      generics_0.1.2      
## [34] farver_2.1.0         jsonlite_1.8.0       distributional_0.3.1
## [37] magrittr_2.0.3       Rcpp_1.0.8.3         munsell_0.5.0       
## [40] fansi_1.0.3          lifecycle_1.0.1      stringi_1.7.6       
## [43] yaml_2.3.5           snakecase_0.11.0     plyr_1.8.7          
## [46] grid_4.2.0           crayon_1.5.1         lattice_0.20-45     
## [49] haven_2.5.0          splines_4.2.0        hms_1.1.1           
## [52] pillar_1.7.0         boot_1.3-28          stats4_4.2.0        
## [55] reshape2_1.4.4       codetools_0.2-18     reprex_2.0.1        
## [58] glue_1.6.2           evaluate_0.15        modelr_0.1.8        
## [61] vctrs_0.4.1          nloptr_2.0.3         tzdb_0.3.0          
## [64] cellranger_1.1.0     gtable_0.3.0         assertthat_0.2.1    
## [67] xfun_0.31            janitor_2.1.0        broom_0.8.0         
## [70] nlmeU_0.70-9         statmod_1.4.37       diagonals_6.4.0     
## [73] ellipsis_0.3.2
```

# Load data

```
  ## Set exclusion parameters
  max_caredays = 90
  min_hosp_mvo_obs = 100

  # Load data if no parsed data file exists
if(file.exists("final_data.Rda")){
  load("final_data.Rda")
  enheter <- read_csv("./enheter.csv")
}else{
         

#Setup for parallel processing

cluster <- new_cluster(8)
# Add needed functions to cluster instances
cluster_copy(cluster,c("first","last","ymd","n","paste","unique","difftime","lead","lag","str_split","across"))

# Get unit/region names for first and last year of study (Reference lists available from NBHW at: https://www.socialstyrelsen.se/utveckla-verksamhet/e-halsa/klassificering-och-koder/andra-kodverk/sjukhuskoder/)

enheter2019 <- read_excel("./kodlistor/sjukhus-och-klinikkoder-for-sluten-vard-2019-patientregistret.xlsx", 
                          sheet = "Slutenvård",
                          col_names = c("namn","mvo","antal")) %>%
  mutate(region = ifelse(is.na(mvo),
                         namn,NA),
         hosp_id = ifelse(!is.na(mvo),
                         word(namn,1,sep = "-"),NA),
         hosp_name = ifelse(!is.na(mvo),
                          word(namn,2,sep = "-"),NA),
         n_2019 = as.numeric(antal)) %>%
  fill(region,hosp_id,hosp_name) %>%
  filter(!is.na(mvo)) %>%
  separate(mvo,c("mvo_id","mvo_namn"),"-",extra = "merge") %>%
  select(-namn,-antal)

enheter2015 <- read_excel("./kodlistor/sjukhus-och-klinikkoder-for-sluten-vard-2015-patientregistret.xlsx", 
                          sheet = "Slutenvård",
                          col_names = c("namn","mvo","antal")) %>%
  mutate(region = ifelse(is.na(mvo),
                         namn,NA),
         hosp_id = ifelse(!is.na(mvo),
                          word(namn,1,sep = "-"),NA),
         hosp_name = ifelse(!is.na(mvo),
                            word(namn,2,sep = "-"),NA),
         n_2015 = as.numeric(antal)) %>%
  fill(region,hosp_id,hosp_name) %>%
  filter(!is.na(mvo)) %>%
  separate(mvo,c("mvo_id","mvo_namn"),"-",extra = "merge") %>%
  select(-namn,-antal) %>%
  filter(!(hosp_id %in% enheter2019$hosp_id & mvo_id %in% enheter2019$mvo_id))

enheter <- bind_rows(enheter2015,enheter2019)

write.csv(enheter,"./enheter.csv",row.names = F)

### Parse inpatient care registry

sv_raw <- read.delim("./NBHW/T_S_R_PAR_SV_5102_2020.txt")

sv <- sv_raw  %>%
  unite(diagnoser, dia1:dia30,sep = " ") %>%
  arrange(lopnr,UTDATUMA) %>%
  #head(10000) %>%
  mutate(same = lopnr == lag(lopnr,default = F),
         # Here we encode the assumption that if the admission date of the next ward contact is the same or prior to (Sometimes there is a delay in writing discharge notes) the discharge date of the previous ward contact, and the admission type is calssified as a transfer from another ward (or is missing), it is part of the same hospital contact.
         cont = INDATUMA <= lag(UTDATUMA,default = F) & (is.na(INSATT) | INSATT == "1"),
         grp = ifelse(same,NA,1),
         diagnoser = trimws(diagnoser)) %>%
  group_by(lopnr) %>%
  mutate(row = row_number())

remove(sv_raw)

# combine transfers between wards into single observations

i=1
while(sum(is.na(sv$grp)) > 0){
  
  lag_grp = lag(sv$grp,default = 1)
  new_grp = ifelse(sv$cont,lag_grp,lag_grp+1)
  
  sv$grp[is.na(sv$grp)] <- new_grp[is.na(sv$grp)]

  print(i)
  i = i +1
}

# Collect ward visits to combined care episodes (parallellized for speed)

sv_vtf <- sv %>%
  #head(10000) %>%
  group_by(lopnr) %>%
  arrange(UTDATUMA) %>%
  # Especially during the first year of data, there are large numbers of missing values for country of birth. Since individuals tend to appear in the data later, we can at least impute these values.. Turns out it's not sufficient though!
  mutate(FLAND = ifelse(FLAND == "Uppgift Saknas",last(FLAND),FLAND)) %>%
  group_by(lopnr,grp) %>%
  partition(cluster = cluster) %>%
  summarize(muni = ifelse(is.na(first(LK)),first(LKF),first(LK)),
            gender = first(KON)-1,
            age = first(ALDER),
            first_hosp = first(SJUKHUS),
            last_hosp = last(SJUKHUS),
            n_enheter = n(),
            mvo = paste(MVO,collapse = " "),
            mvo_last = last(MVO),
            drg_last = last(drg),
            drg = paste(unique(drg),collapse = " "),
            in_date = ymd(min(INDATUMA)),
            out_date = ymd(max(UTDATUMA)),
            source_admit = first(INSATT),
            source_discharge = last(UTSATT),
            planned = paste(PVARD,collapse = " "),
            diag_prim = paste(unique(HDIA),collapse = " "),
            diag_prim_last = last(HDIA),
            diag_sec = paste(trimws(diagnoser),collapse = " "),
            interventions = paste(unique(trimws(OP)),collapse = " "),
            born = first(FLAND),
            civil_status = first(CIVIL),
            drg = paste(unique(drg),collapse = " ")) %>%
  collect() %>% 
  mutate(caredays = as.numeric(round(difftime(out_date,in_date,units="days")))+1,
         diag_sec = trimws(sapply(strsplit(diag_sec, " "), function(x) paste(unique(x), collapse = " "))),
         interventions = trimws(sapply(strsplit(interventions, " "), function(x) paste(unique(x), collapse = " "))),
         n_diag = sapply(str_split(diag_sec," "),length),
         n_op = sapply(str_split(interventions," "),length),
         n_diag = ifelse(diag_sec == "",0,n_diag),
         n_op = ifelse(interventions == "",0,n_op)) %>%
  left_join(distinct(select(enheter,last_hosp = hosp_id,hosp_name,region)),by = "last_hosp")

# Load cause of death registry
dors_raw <- read.delim("./NBHW/R_DORS__5102_2020.txt") %>%
  select(-AR) %>%
  mutate(death_date = ymd(DODSDAT))

# Load ambulatory care registry data

ov_raw <- read.delim(".//NBHW/S_R_PAR_OV_5102_2020.txt") %>%
  select(-AR) %>%
  mutate(date = ymd(INDATUMA))

# Load social care registry data

hsl <- read.delim("./NBHW/R2_HSL__5102_2020.txt") %>%
  select(-SURV,-DATAVREG) %>%
  pivot_longer(JAN:DEC) %>%
  filter(!is.na(value)) %>%
  mutate(name = ifelse(name == "OKT","OCT",name),
         name = ifelse(name == "MAJ","MAY",name),
         date_month = ymd(paste(AR,name,"01"))) %>%
  distinct()

sol_incr <- read.delim("./NBHW/R2_SOL_INCR_5102_2020.txt") %>%
  group_by(lopnr,PERIOD,AR) %>%
  summarise(across(.fns = max))

sol <- read.delim("./NBHW/R2_SOL__5102_2020.txt") %>%
  mutate(date_month = ymd(paste(PERIOD,"01"))) %>%
  select(lopnr,date_month,HTJ,TRYGG,KORTTID,ABIST) %>%
  mutate(ABIST = ifelse(ABIST == 9,NA,ABIST),
         KORTTID = ifelse(KORTTID == 9,NA,KORTTID)) %>%
  full_join(select(hsl,lopnr,date_month,HSL = value),by = c("lopnr","date_month")) %>%
  mutate(across(HTJ:HSL,replace_na,0)) %>%
  group_by(lopnr,date_month) %>%
  partition(cluster = cluster) %>%
  summarise(across(HTJ:HSL,max)) %>%
  collect()

# define ACSC conditions

# We didn't wind up using this other than in the case-mix adjustment models, but oh well!

# Per the definition of "ACSCs in common use in the NHS" in https://doi.org/10.1016/j.puhe.2008.11.001
purdy_icd_str = "I20|I240|I248|I249|J45|J46|L03|L04|L080|L088|L089|L88|L980|I110|I50|J81|G40|G41|R56|O15|J20|J41|J42|J43|J47|E86|K522|K528|K529|A690|K02|K03|K04|K05|K06|K08|K098|K099|K12|K13|E10[0-8]|E11[0-8]|E12[0-8]|E13[0-8]|E14[0-8]|H66|H67|J02|J03|J06|J312|R02|I10|I119|J10|J11|J13|J14|J153|J154|J157|J159|J168|J181|J18|D501|D508|D509|E40|E41|E42|E43|E550|E643|A35|A36|A37|A80|B05|B06|B161|B169|B180|B181|B26|G000|M014|N70|N73|N74|K25[0-2]|K25[4-6]|K26[0-2]|K26[4-6]|K27[0-2]|K27[4-6]|K28[0-2]|K28[4-6]|N10|N11|N12|N136"

# Per the definition of indicator 1 in https://www.socialstyrelsen.se/globalassets/sharepoint-dokument/artikelkatalog/ovrigt/2014-2-12.pdf

nbhw_icd_str = "I50|N39|N109|N309|J09|J10|J11|J13|J14|J15|J16|J17|J18|J44|J45|J46|I20|I24"

nbhw_icd_kol_p_str = "J20"
nbhw_icd_kol_s_str = "J41|J42|J43|J44|J47"

nbhw_icd_diab_s_str =  "E10[1-8]|E11[1-8]|E13[1-8]|E14[1-8]"

# Calculate outcome measures

d <- sv_vtf %>%
  #head(10000)  %>%
  left_join(dors_raw,by="lopnr") %>%
  arrange(lopnr,in_date) %>%
  mutate(acsc_purdy = ifelse(grepl(purdy_icd_str,diag_prim_last),1,0),
         acsc_nbhw = ifelse(grepl(nbhw_icd_str,diag_prim_last)|
                              (grepl(nbhw_icd_kol_p_str,diag_prim_last) & grepl(nbhw_icd_kol_s_str,diag_sec))|
                              grepl(nbhw_icd_diab_s_str,diag_sec),1,0)) %>%
  group_by(lopnr) %>%
  partition(cluster = cluster) %>%
  mutate(next_admit = round(difftime(lead(in_date),out_date,units="days")),
         next_admit_days = lead(out_date) - lead(in_date),
         next_planned = ifelse(lead(substr(planned,1,1)) == "1",T,F),
         next_acsc_purdy = ifelse(lead(acsc_purdy) == 1,T,F),
         next_acsc_nbhw = ifelse(lead(acsc_nbhw) == 1,T,F)) %>%
  collect() %>%
  ungroup() %>%
# note here, per CMS definition, readmissions after a planned readmission within 30 days are not counted as a positive outcome:
# https://www.cms.gov/Medicare/Medicare-Fee-for-Service-Payment/PhysicianFeedbackProgram/Downloads/2015-ACR-MIF.pdf
  mutate(readmit30 = ifelse(next_admit <= 30 & !is.na(next_admit),1,0),
         unplanreadmit30 = ifelse(next_admit <= 30 & !is.na(next_admit) & !next_planned,1,0),
         mort_days = difftime(ymd(DODSDAT),out_date,units="days"),
         mort30 = ifelse(mort_days <= 30 & !is.na(mort_days),1,0),
         discharge_date_month = floor_date(ymd(out_date),unit = "months"),
         SABO = ifelse(source_discharge == 2,1,0)) %>%
  left_join(sol,
            by = c("lopnr" = "lopnr",
                   "discharge_date_month" = "date_month")) %>%
  mutate(across(HTJ:HSL,replace_na,0),
         any_social = ifelse(SABO+HTJ+TRYGG+KORTTID+ABIST+HSL == 0 & !is.na(SABO),0,1),
         id = paste(lopnr,grp,sep="_")) %>%
  arrange(id)

# Add ambulatory care visits - The best solution for getting date overlaps seems to be the foverlaps function from data.table. Otherwise I try to stick to the tidyverse.

t <- d %>%
  mutate(out_date_followup = out_date + 30)

t_ov <- setDT(ov_raw)
t_ov[,date_copy := copy(date)]
setkey(t_ov, lopnr, date, date_copy)

setDT(t)
setkey(t, lopnr, out_date, out_date_followup)

# fast overlaps
ov_nest = foverlaps(t, t_ov,
                    by.x = c("lopnr", "out_date", "out_date_followup"),
                    by.y = c("lopnr", "date", "date_copy")) %>%
  group_by(lopnr,grp,PVARD) %>%
  partition(cluster = cluster) %>%
  summarise(first_ov_date = first(date)) %>%
  collect() %>%
  pivot_wider(names_from = PVARD,values_from = first_ov_date) %>%
  select(-'NA',ov_planned_date = '1',ov_unplanned_date = '2')

d <- d %>%
  left_join(ov_nest)

# recode civil status - The NBHW codes marriages and civil partnerships seperately, but there are so few civil partnerships in the data that it only clutters the analysis... We go ahead and group them together
d$civil_status[d$civil_status %in% c("G","RP")] <- "Married"
d$civil_status[d$civil_status == "OG"] <- "Unmarried"
d$civil_status[d$civil_status %in% c("S","SP")] <- "Divorced"
d$civil_status[d$civil_status %in% c("Ä","EP")] <- "Widow"
d$civil_status[d$civil_status %in% c("")] <- NA


#Recode place of birth - Again, very few from each of the regions outside of europe that they're not worth analyzing seperately

d$born[d$born == "Sverige"] <- "Sweden"
d$born[d$born == "Övriga Norden"] <- "Nordics"
d$born[d$born == "Europa"] <- "Rest of Europe"
d$born[d$born %in% c("Afrika","Asien","Nordamerika","Oceanien","Sovjetunionen","Sydamerika","Övrigt")] <- "Outside Europe"
d$born[d$born == "Uppgift Saknas"] <- NA

# Some final processing to form the dataset of included patients to make it more analysis friendly

d <- d %>%
  mutate(in_date = ymd(in_date),
         out_date = ymd(out_date),
         since_start = elapsed_months(out_date,ymd("20150101")),
         post_intervention = ifelse(out_date>=ymd("20180101"),
                                    1,0),
         since_intervention = ifelse(out_date>=ymd("20180101"),
                                     elapsed_months(out_date,ymd("20180101")),0),
         month_dummy = factor(month(out_date)),
         civil_status = relevel(factor(civil_status),ref = "Married"),
         born = relevel(factor(born), ref = "Sweden"),
         any_social = ifelse(is.na(any_social),0,any_social),
         social_fct = relevel(factor(ifelse(any_social == 0,
                                            "None",ifelse(SABO == 0, "in_home","nursing_home"))),ref="None"),
         planned_contact = ifelse(substr(planned,1,1) == "1",
                                  1,0),
         ovunplanned_days = ovunplanned_date - out_date,
         ovunplanned = ifelse(!is.na(ovunplanned_days) & 
                                 (ovunplanned_days <= next_admit | 
                                    is.na(next_admit)),1,0),
         ovunplanned_30 = ifelse(!is.na(ovunplanned_days) &
                                 ovunplanned_days <=30 &
                                 (ovunplanned_days <= next_admit | 
                                    is.na(next_admit)),1,0),
         ovplanned_days = ovplanned_date - out_date,
         ovplanned_30 = ifelse(!is.na(ovplanned_days) & 
                                 ovplanned_days <=30 &
                               (ovplanned_days <= next_admit |
                                  is.na(next_admit)),
                             1,0),
         last_hosp_mvo = paste(last_hosp,mvo_last,sep = "_")) %>%
  # Note that up until this point, in-patient stays with only a single ICD codes have been included in the dataset despite not meeting our inclusion criteria in order to calculate valid re-admission rates. Here we can finally remove them.
  
# A further note regarding ambulatory care visits: we had specified in our analysis plan that we would investigate "Emergency" ambulatory care visits - However this variable was not tracked prior to 2016. We found that unplanned and emergency contacts mapped very well - 94% of unplanned contacts were, in fact, emergency contacts of some kind. Thus, rather than sacrifice a year of data we used this as a proxy instead.
  filter(n_diag >= 2) %>%
  group_by(last_hosp_mvo) %>%
  mutate(n_hosp_mvo = n(),
         last_hosp_mvo = ifelse(n_hosp_mvo>=min_hosp_mvo_obs,
                                as.character(last_hosp_mvo),NA)) %>%
  ungroup() %>%
  mutate(unplanreadmit30_acsc_neg = ifelse(unplanreadmit30 & 
                                             !next_acsc_purdy == 1,1,0),
         unplanreadmit30_acsc_purdy = ifelse(unplanreadmit30 & 
                                             next_acsc_purdy == 1,1,0),
         pct_unplanreadmit30_acsc = ifelse(unplanreadmit30,
                                           ifelse(next_acsc_purdy == 1,1,0),
                                           NA)) %>%
  ungroup() %>%
  mutate(unplanreadmitdays = ifelse(is.na(next_admit_days) | next_admit_days > max_caredays,
                                    max_caredays,
                                    next_admit_days),
         unplanreadmitcensor = ifelse(is.na(next_admit_days) | next_planned | next_admit_days > max_caredays,
                                      1,0),
         unplanreadmit7 = ifelse(unplanreadmit30 & next_admit_days <= 7,
                                 1,0),
         v=1) %>%
  arrange(id)

  save(d,file = "final_data.Rda")
  
}
```

```
## Rows: 1143 Columns: 7
## ── Column specification ────────────────────────────────────────────────────────
## Delimiter: ","
## chr (5): mvo_id, mvo_namn, region, hosp_id, hosp_name
## dbl (2): n_2015, n_2019
## 
## ℹ Use `spec()` to retrieve the full column specification for this data.
## ℹ Specify the column types or set `show_col_types = FALSE` to quiet this message.
```

```
# Apply exclusion criteria, and save the number of included records at each step.
excl_n <- list()

excl_n$orig <- nrow(d)

d <- d %>%
  filter(!is.na(source_discharge),
         !is.na(source_admit),
         !is.na(last_hosp))

excl_n$missingdata <- nrow(d)

d <- d %>%
  filter(!source_discharge %in% c(4))

excl_n$discharge <- nrow(d)

d <- d %>%
  filter(caredays <= max_caredays)

excl_n$longstay <- nrow(d)

d <- d %>%
  filter(out_date < ymd("20200101") - days(max_caredays))

excl_n$endofstudy <- nrow(d)

d <- d %>%
  filter(!is.na(last_hosp_mvo))

excl_n$final <- nrow(d)

if(file.exists("grouped_data.csv")){
  d_grp <- read_csv("grouped_data.csv") %>%
    mutate(month_dummy = as.factor(month_dummy))
}else{
  
  
  # Generate aggregate dataset
d_grp <- d %>%
  bind_cols(pivot_wider(d,id_cols = id,
                        names_from = civil_status,
                        names_glue = "status_{civil_status}",
                        values_from = v,
                        values_fill = 0)) %>%
  bind_cols(pivot_wider(d,id_cols = id,
                        names_from = born,
                        names_glue = "born_{born}",
                        values_from = v,
                        values_fill = 0)) %>%
  bind_cols(pivot_wider(d,id_cols = id,
                        names_from = social_fct,
                        names_glue = "soc_{social_fct}",
                        values_from = v,
                        values_fill = 0)) %>%
  dplyr::select(out_date,
                caredays, 
               unplanreadmit30,
               readmit30,
               mort30,
               ovplanned_30,
               ovunplanned_30,
               since_start, 
               post_intervention, 
               since_intervention,  
               age, 
               gender, 
               starts_with("status_"), 
               starts_with("born_"), 
               starts_with("soc_"),
               planned_contact, 
               n_diag, 
               n_op, 
               acsc_purdy, 
               any_social) %>%
  mutate(population = ifelse(any_social == 1,"Social care","No social care")) %>%
  bind_rows(mutate(., population = "All")) %>%
  group_by(out_date = floor_date(out_date,unit = "month"),population) %>%
  mutate(n = n()) %>%
  summarise(across(.cols = everything(),.fns = mean,na.rm=T)) %>%
  ungroup() %>%
  mutate(month_dummy = factor(month(out_date))) %>%
  filter(since_start < 57) # Note: Since the inclusion criteria is technically the end of 2019 minus 90 days, two days of October are included.. We'll remove these from the grouped dataset so we don't need to deal with a month containing only a (relatively) small number of observations

names(d_grp) <- make.names(names(d_grp))

write_csv(d_grp,file = "grouped_data.csv")

}
```

```
## Rows: 171 Columns: 33
## ── Column specification ────────────────────────────────────────────────────────
## Delimiter: ","
## chr   (1): population
## dbl  (31): caredays, unplanreadmit30, readmit30, mort30, ovplanned_30, ovunp...
## date  (1): out_date
## 
## ℹ Use `spec()` to retrieve the full column specification for this data.
## ℹ Specify the column types or set `show_col_types = FALSE` to quiet this message.
```

# Estimate models

```
# Note that I've cut out a bunch of messy code here relating to model assumption checking, evaluating clustering effects at various levels, interim visualizations, etc. If you're interested in seeing something along those lines, get in touch and I'll try to get something put together for you!

# Another note: We're doing something here that I normally don't like: Applying linear models to non-multivariate normal data. 30-day readmission rates are obviously binomially distributed. Length of stay is trickier to nail down, and there are a number of articles investigating the fit of various distributions to length of stay data. A gamma or overdispersed poisson distribution for example fits our data reasonably well. Many of the misspecification issues that arise from this are most problematic in small sample sizes, and these linear models do appear to generate valid point estimates which are perhaps even more interpretable for non-statisticians than the odds- and hazard ratios that would be generated by their generalized counterparts. While GLMMs are arguably more appropriate, the sample size, number of parameters in the controlled models, and multiple levels of clustering we need to account for results in such models simply never converging (at least they don't with the estimators we've tried over the several days they've been allowed to run). So - We instead let our model residuals be skewed and bootstrap our way out of the problem of generating valid confidence intervals for interpreting significance. This should be considered when interpreting our secondary findings which here are reported using the standard Wald t-values provided by the lme4 summary function - The bootstrapped CIs are generally a bit more conservative than variance estimates based on distributional assumptions.

# One more note: These models converge slowly, and some wind up exceeding the default lme4 gradient tolerances, though all models have a max gradient <= 0.0053 by the end of the fitting process, which seems acceptable.

# Define final models

if(file.exists("final_models.Rda")){
  
  load("final_models.Rda")
  load("final_models_sec.Rda")
  
  }else{
    
    mod_list <- list()
    # Base models
    print(Sys.time())
    mod_list$caredays <- lmer(caredays ~ 
                           since_start + 
                           post_intervention + 
                           since_intervention +
                         (1|last_hosp_mvo) +
                         (1|month_dummy),
                         data = d)

    print(Sys.time())
    mod_list$readmit <- lmer(unplanreadmit30 ~ 
                          since_start + 
                          post_intervention + 
                          since_intervention + 
                          (1|last_hosp_mvo) +
                          (1|month_dummy), 
                        data = d)
  
  #Control series
  print(Sys.time())
  mod_list$caredays_soc <- lmer(caredays ~ 
                           (since_start + 
                            post_intervention + 
                            since_intervention) * any_social +
                           (1|last_hosp_mvo) +
                           (1|month_dummy),
                           data = d)
  
  print(Sys.time())
  mod_list$readmit_soc <- lmer(unplanreadmit30 ~ 
                          (since_start + 
                             post_intervention + 
                             since_intervention) * any_social + 
                          (1|last_hosp_mvo) +
                          (1|month_dummy),
                          data = d)
  
  # Risk adjusted
  print(Sys.time())
  mod_list$caredays_adj <- lmer(caredays ~ 
                                since_start + 
                                post_intervention + 
                                since_intervention + 
                                age + 
                                gender + 
                                civil_status +
                                planned_contact +
                                n_diag +
                                n_op +
                                acsc_purdy +
                                social_fct +
                                (1|last_hosp_mvo) +
                                (1|month_dummy),
                                data = d)
  
  print(Sys.time())
  mod_list$readmit_adj <- lmer(unplanreadmit30 ~
                               since_start + 
                               post_intervention + 
                               since_intervention + 
                               age + 
                               gender + 
                               civil_status +
                               planned_contact +
                               n_diag +
                               n_op +
                               acsc_purdy +
                               social_fct +
                               (1|last_hosp_mvo) +
                               (1|month_dummy),
                               data = d)
  print(Sys.time())
  
  mod_list_sec <- list()
  
  mod_list_sec$mortality <- lmer(mort30 ~ 
                        since_start + 
                        post_intervention + 
                        since_intervention + 
                        (1|last_hosp_mvo) +
                        (1|month_dummy), 
                      data = d)

  print(Sys.time())
  
  mod_list_sec$mortality_soc <- lmer(mort30 ~ 
                            (since_start + 
                             post_intervention + 
                             since_intervention) * any_social + 
                            (1|last_hosp_mvo) +
                            (1|month_dummy),
                      data = d)

  mod_list_sec$ovplanned <- lmer(ovplanned_30 ~ 
                         since_start + 
                         post_intervention + 
                         since_intervention + 
                         (1|last_hosp_mvo) +
                         (1|month_dummy), 
                      data = d)

  print(Sys.time())

  mod_list_sec$ovplanned_soc <- lmer(ovplanned_30 ~ 
                         (since_start + 
                          post_intervention + 
                          since_intervention) * any_social + 
                         (1|last_hosp_mvo) +
                         (1|month_dummy), 
                      data = d)

  print(Sys.time())
  
  mod_list_sec$ovunplanned <- lmer(ovunplanned_30 ~ 
                           since_start + 
                           post_intervention + 
                           since_intervention + 
                           (1|last_hosp_mvo) +
                           (1|month_dummy), 
                      data = d)

  print(Sys.time())
  
  mod_list_sec$ovunplanned_soc <- lmer(ovunplanned_30 ~ 
                         (since_start + 
                          post_intervention + 
                          since_intervention) * any_social + 
                         (1|last_hosp_mvo) +
                         (1|month_dummy), 
                      data = d)

  print(Sys.time())
  
    mod_list_sec$mortality_adj <- lmer(mort30 ~ 
                          since_start + 
                          post_intervention + 
                          since_intervention +  
                          age + 
                          gender + 
                          civil_status +
                          planned_contact +
                          n_diag +
                          n_op +
                          acsc_purdy +
                          social_fct +
                          (1|last_hosp_mvo) +
                          (1|month_dummy),
                          data = d)
  print(Sys.time())
  
    mod_list_sec$ovunplanned_adj <- lmer(ovunplanned_30 ~ 
                          since_start + 
                          post_intervention + 
                          since_intervention +  
                          age + 
                          gender + 
                          civil_status +
                          planned_contact +
                          n_diag +
                          n_op +
                          acsc_purdy +
                          social_fct +
                          (1|last_hosp_mvo) +
                          (1|month_dummy),
                          data = d)
  print(Sys.time())
  
    mod_list_sec$ovplanned_adj <- lmer(ovplanned_30 ~ 
                          since_start + 
                          post_intervention + 
                          since_intervention +  
                          age + 
                          gender + 
                          civil_status +
                          planned_contact +
                          n_diag +
                          n_op +
                          acsc_purdy +
                          social_fct +
                          (1|last_hosp_mvo) +
                          (1|month_dummy),
                          data = d)
    
  print(Sys.time())
  
  mod_list_sec$caredays_soc_fct <- lmer(caredays ~ 
                         (since_start + post_intervention + since_intervention) * social_fct + 
                         (1|last_hosp_mvo) +
                         (1|month_dummy),
                         data = d)
  
  print(Sys.time())
  
  mod_list_sec$readmit_soc_fct <- lmer(unplanreadmit30 ~ 
                        (since_start + post_intervention + since_intervention) * social_fct + 
                        (1|last_hosp_mvo) +
                        (1|month_dummy), 
                        data = d)
  
  print(Sys.time())
  mod_list_sec$caredays_soc_adj <- lmer(caredays ~
                           (since_start + 
                            post_intervention + 
                            since_intervention) * any_social + 
                           age + 
                           gender + 
                           civil_status +
                           planned_contact +
                           n_diag +
                           n_op +
                           acsc_purdy +
                           (1|last_hosp_mvo) +
                           (1|month_dummy),
                           data = d)
  
  print(Sys.time())
  mod_list_sec$readmit_soc_adj <- lmer(unplanreadmit30 ~ 
                          (since_start + 
                            post_intervention + 
                            since_intervention) * any_social +
                          age + 
                          gender + 
                          civil_status +
                          planned_contact +
                          n_diag +
                          n_op +
                          acsc_purdy +
                          (1|last_hosp_mvo) +
                          (1|month_dummy),
                          data = d)
  
  mod_list_sec$readmit_all <- lmer(readmit30 ~ 
                          since_start + 
                          post_intervention + 
                          since_intervention + 
                          (1|last_hosp_mvo) +
                          (1|month_dummy), 
                        data = d)
  
  mod_list_sec$readmit_all_soc <- lmer(readmit30 ~ 
                          (since_start + 
                             post_intervention + 
                             since_intervention) * any_social + 
                          (1|last_hosp_mvo) +
                          (1|month_dummy),
                          data = d)
  
  print(Sys.time())
  mod_list_sec$readmit_all_adj <- lmer(readmit30 ~ 
                                since_start + 
                                post_intervention + 
                                since_intervention + 
                                age + 
                                gender + 
                                civil_status +
                                planned_contact +
                                n_diag +
                                n_op +
                                acsc_purdy +
                                social_fct +
                                (1|last_hosp_mvo) +
                                (1|month_dummy),
                                data = d)
  
  save(mod_list,file = "final_models.Rda")
  save(mod_list_sec,file = "final_models_sec.Rda")
  
  }
```

# Calculate bootstrap CIs

```
if(file.exists("final_boot.Rda")){
  
  load("final_boot.Rda")
    
}else{
  
  set.seed(42)
  
  #Setup cluster instances for foreach which, of course, uses a different paralellization scheme (doParallel) than multidbplyr.
  no_cores <- 7
  cl <- makeCluster(no_cores)
  
  registerDoParallel(cores = no_cores)
  
  # Run 143 bootstrap iterations on each core (1001 total). Note that this can take multiple days to run.
  
  boot_list <- list()
  for (i in 1:length(mod_list)){
    print(Sys.time())
    print(names(mod_list[i]))
    boot_list[names(mod_list[i])] <- list(boot_parallel(mod_list[[i]],cl))
    gc()
  }

  save(boot_list,file="final_boot.Rda")
  
}
```

# Tables and figures

## Figure 2 - Aggregate data desription

```
fig2_full <- d %>%
  mutate(date = ymd(out_date)) %>%
  group_by(date) %>%
  summarise("Number of daily admissions" = n(),
            "Average age" = mean(age),
            "Percent female" = mean(gender),
            "Percent planned admissions" = mean(planned_contact),
#            "Average number of diagnoses" = mean(n_diag),
#            "Average number of interventions" = mean(n_op),
            "Percent social care" = mean(any_social),
            "Percent 30-day mortality" = mean(mort30,na.rm = T),
            "Percent 30-day planned ambulatory care" = mean(ovplanned_30,na.rm = T),
            "Percent 30-day unplanned ambulatory care" = mean(ovunplanned_30,na.rm = T),
            "Average care days"= mean(caredays,na.rm = T),
            "Percent 30-day unplanned readmissions" = mean(unplanreadmit30,na.rm = T)
            ) %>%
  gather("key","value",-date) %>%
  group_by(key) %>%
  mutate(rollmean = rollmean(value, 28, na.pad=TRUE)) %>%
  ungroup() %>%
  mutate(key = factor(key, levels = unique(key)[c(2,3,1,4,5,6,7,8,9,10)]),
         group = "Full sample")


fig2_data <- d %>%
  mutate(date = ymd(out_date)) %>%
  group_by(date,any_social) %>%
  summarise("Number of daily admissions" = n(),
            "Average age" = mean(age),
            "Percent female" = mean(gender),
            "Percent planned admissions" = mean(planned_contact),
#            "Average number of diagnoses" = mean(n_diag),
#            "Average number of interventions" = mean(n_op),
            "Percent social care" = mean(any_social),
            "Percent 30-day mortality" = mean(mort30,na.rm = T),
            "Percent 30-day planned ambulatory care" = mean(ovplanned_30,na.rm = T),
            "Percent 30-day unplanned ambulatory care" = mean(ovunplanned_30,na.rm = T),
            "Average care days"= mean(caredays,na.rm = T),
            "Percent 30-day unplanned readmissions" = mean(unplanreadmit30,na.rm = T)
            ) %>%
  gather("key","value",-date,-any_social) %>%
  group_by(key,any_social) %>%
  mutate(rollmean = rollmean(value, 28, na.pad=TRUE)) %>%
  ungroup() %>%
  mutate(key = factor(key, levels = unique(key)[c(2,3,1,4,5,6,7,8,9,10)]),
         group = ifelse(any_social == 0,"No social care","Social care")) %>%
  bind_rows(fig2_full) %>%
  filter(rollmean != 0 & rollmean != 1)
```

```
## `summarise()` has grouped output by 'date'. You can override using the
## `.groups` argument.
```

```
fig2 <- fig2_data %>%
  ggplot(aes(x=date,y=rollmean,color=group,group=group)) +
  geom_line(size=1) +
  #geom_smooth(span = 0.5,se = F) +
  geom_vline(xintercept = ymd("20180101"),color = "red", size = 1) +
  scale_color_manual(values = c("black","blue","darkgreen")) +
  #scale_linetype_manual(values = c("solid","dashed","dashed")) +
  #expand_limits(y = 0) +
  facet_wrap(~key,scales = "free_y",ncol = 2) +
  theme(panel.grid.minor = element_blank()) +
  labs(y="4-week rolling mean value")
 
 
fig2
```

```
# Save as high-res tiff

# tiff("fig2.tif", res=300,height = 8,width = 8,units = "in")
# fig2
# dev.off()
```

## Figure 3 - Model summaries

```
getmode <- function(v) {
   uniqv <- unique(v)
   uniqv[which.max(tabulate(match(v, uniqv)))]
}

modes <- d %>%
  summarise(across(everything(),getmode))

d_grp_data <- d_grp %>% 
  dplyr::select(since_start,
         post_intervention,
         since_intervention,
         month_dummy,
         caredays,
         unplanreadmit30,
         any_social) %>%
  bind_cols(dplyr::select(modes,-which((names(modes) %in% names(.)))))

d_grp_data_cf <- d_grp_data %>%
  mutate(post_intervention = 0,
         since_intervention = 0)

d_grp_data_social0 <- mutate(d_grp_data,any_social = 0)
d_grp_data_social1 <- mutate(d_grp_data,any_social = 1)

d_grp_data_social0_cf <- mutate(d_grp_data_cf,any_social = 0)
d_grp_data_social1_cf <- mutate(d_grp_data_cf,any_social = 1)


d_grp_preds <- data.frame(rbind(cbind("month"=d_grp_data$since_start,
                                      "fw"="ITS",
                                      "out"="Inpatient care\nduration (days)",
                                      "grp"="Full sample",
                                      "key"="Pre-intervention trend",
                                      "value"=predict(mod_list$caredays,
                                                      newdata = d_grp_data_cf,
                                                      type = "response",
                                                      re.form = NA)),
                                cbind("month"=d_grp_data$since_start,
                                      "fw"="ITS",
                                      "out"="30-day unplanned\nreadmission (%)",
                                      "grp"="Full sample",
                                      "key"="Pre-intervention trend",
                                      "value"=predict(mod_list$readmit,
                                                      newdata = d_grp_data_cf,
                                                      type = "response",
                                                      re.form = NA)),
                                cbind("month"=d_grp_data$since_start,
                                      "fw"="ITS",
                                      "out"="Inpatient care\nduration (days)",
                                      "grp"="Full sample",
                                      "key"="Post-intervention",
                                      "value"=predict(mod_list$caredays,
                                                      newdata = d_grp_data,
                                                      type = "response",
                                                      re.form = NA)),
                                cbind("month"=d_grp_data$since_start,
                                      "fw"="ITS",
                                      "out"="30-day unplanned\nreadmission (%)",
                                      "grp"="Full sample",
                                      "key"="Post-intervention",
                                      "value"=predict(mod_list$readmit,
                                                      newdata = d_grp_data,
                                                      type = "response",
                                                      re.form = NA)),
                                
                                
                                
                                # Control groups
                                
                                cbind("month"=d_grp_data$since_start,
                                      "fw"="CITS",
                                      "out"="Inpatient care\nduration (days)",
                                      "grp"="Social care",
                                      "key"="Post-intervention",
                                      "value"=predict(mod_list$caredays_soc,
                                                      newdata = d_grp_data_social1,
                                                      type = "response",
                                                      re.form = NA)),
                                
                                cbind("month"=d_grp_data$since_start,
                                      "fw"="CITS",
                                      "out"="30-day unplanned\nreadmission (%)",
                                      "grp"="Social care",
                                      "key"="Post-intervention",
                                      "value"=predict(mod_list$readmit_soc,
                                                      newdata = d_grp_data_social1,
                                                      type = "response",
                                                      re.form = NA)),
                                cbind("month"=d_grp_data$since_start,
                                      "fw"="CITS",
                                      "out"="Inpatient care\nduration (days)",
                                      "grp"="Social care",
                                      "key"="Pre-intervention trend",
                                      "value"=predict(mod_list$caredays_soc,
                                                      newdata = d_grp_data_social1_cf,
                                                      type = "response",
                                                      re.form = NA)),
                                
                                cbind("month"=d_grp_data$since_start,
                                      "fw"="CITS",
                                      "out"="30-day unplanned\nreadmission (%)",
                                      "grp"="Social care",
                                      "key"="Pre-intervention trend",
                                      "value"=predict(mod_list$readmit_soc,
                                                      newdata = d_grp_data_social1_cf,
                                                      type = "response",
                                                      re.form = NA)),
                                
                                
                                
                                cbind("month"=d_grp_data$since_start,
                                      "fw"="CITS",
                                      "out"="Inpatient care\nduration (days)",
                                      "grp"="No Social care",
                                      "key"="Post-intervention",
                                      "value"=predict(mod_list$caredays_soc,
                                                      newdata = d_grp_data_social0,
                                                      type = "response",
                                                      re.form = NA)),
                                
                                cbind("month"=d_grp_data$since_start,
                                      "fw"="CITS",
                                      "out"="30-day unplanned\nreadmission (%)",
                                      "grp"="No Social care",
                                      "key"="Post-intervention",
                                      "value"=predict(mod_list$readmit_soc,
                                                      newdata = d_grp_data_social0,
                                                      type = "response",
                                                      re.form = NA)),
                                cbind("month"=d_grp_data$since_start,
                                      "fw"="CITS",
                                      "out"="Inpatient care\nduration (days)",
                                      "grp"="No Social care",
                                      "key"="Pre-intervention trend",
                                      "value"=predict(mod_list$caredays_soc,
                                                      newdata = d_grp_data_social0_cf,
                                                      type = "response",
                                                      re.form = NA)),
                                
                                cbind("month"=d_grp_data$since_start,
                                      "fw"="CITS",
                                      "out"="30-day unplanned\nreadmission (%)",
                                      "grp"="No Social care",
                                      "key"="Pre-intervention trend",
                                      "value"=predict(mod_list$readmit_soc,
                                                      newdata = d_grp_data_social0_cf,
                                                      type = "response",
                                                      re.form = NA)),
                                
                                # Covariate adjusted
                                
                                cbind("month"=d_grp_data$since_start,
                                      "fw"="Case-mix adjusted",
                                      "out"="Inpatient care\nduration (days)",
                                      "grp"="Full sample",
                                      "key"="Pre-intervention trend",
                                      "value"=predict(mod_list$caredays_adj,
                                                      newdata = d_grp_data_cf,
                                                      type = "response",
                                                      re.form = NA)),
                                cbind("month"=d_grp_data$since_start,
                                      "fw"="Case-mix adjusted",
                                      "out"="30-day unplanned\nreadmission (%)",
                                      "grp"="Full sample",
                                      "key"="Pre-intervention trend",
                                      "value"=predict(mod_list$readmit_adj,
                                                      newdata = d_grp_data_cf,
                                                      type = "response",
                                                      re.form = NA)),
                                cbind("month"=d_grp_data$since_start,
                                      "fw"="Case-mix adjusted",
                                      "out"="Inpatient care\nduration (days)",
                                      "grp"="Full sample",
                                      "key"="Post-intervention",
                                      "value"=predict(mod_list$caredays_adj,
                                                      newdata = d_grp_data,
                                                      type = "response",
                                                      re.form = NA)),
                                
                                cbind("month"=d_grp_data$since_start,
                                      "fw"="Case-mix adjusted",
                                      "out"="30-day unplanned\nreadmission (%)",
                                      "grp"="Full sample",
                                      "key"="Post-intervention",
                                      "value"=predict(mod_list$readmit_adj,
                                                      newdata = d_grp_data,
                                                      type = "response",
                                                      re.form = NA))
                                
                                ),
                          stringsAsFactors = F) %>%
  mutate(value = as.numeric(value),
         month = as.numeric(month),
         date = ymd(paste0(2015+floor(month/12),sprintf("%02d",month%%12+1),"01")))


fig3 <- d_grp_preds %>%
  mutate(fw = factor(fw,levels = unique(fw)[c(1,3,2)]),
         Group = factor(grp,levels = unique(grp))) %>%
  ggplot(aes(x=date,y=value,color = paste(key,Group))) +
  geom_vline(xintercept = ymd("20180101"),color = "red",size=1)+
  geom_line(size=1)+
  facet_grid(out~fw,scales = "free_y") + 
  #scale_linetype_manual(values = c(1,1,2)) +
  scale_color_manual(values = c("red","red","red","black","blue","darkgreen")) +
  #scale_x_continuous(breaks = seq(0,60,12)) +
  theme(legend.position="bottom",
        panel.grid.minor = element_blank()) +
  expand_limits(y = 0)
  
fig3
```

```
# Save as high-res tiff

 tiff("fig3.tif", res=300,height = 6,width = 8,units = "in")
 fig3
 dev.off()
```

```
## png 
##   2
```

## Table 1 - Base model coefficients

```
caredays_mod_ci <- confint(boot_list$caredays,type = "perc")
readmit_mod_ci <- confint(boot_list$readmit,type = "perc")

cd_ci <- bind_cols(coef = names(fixef(mod_list$caredays)), 
                   caredays_mod_ci[2:4]) %>%
  mutate(text = paste0(round(estimate,3)," (",round(lower,3)," - ",round(upper,3),")"))

re_ci <- bind_cols(coef = names(fixef(mod_list$readmit)), 
                   readmit_mod_ci[2:4]) %>%
  mutate(text = paste0(round(estimate*100,3)," (",round(lower*100,3)," - ",round(upper*100,3),")"))

t1 <- cbind("Coefficient"=cd_ci$coef,
      "30-day unplanned readmission (%)"=re_ci$text,
      "Inpatient length of stay (days)"=cd_ci$text)

t1
```

```
##      Coefficient          30-day unplanned readmission (%)
## [1,] "(Intercept)"        "19.047 (18.642 - 19.382)"      
## [2,] "since_start"        "-0.017 (-0.03 - -0.004)"       
## [3,] "post_intervention"  "0.571 (0.108 - 1.039)"         
## [4,] "since_intervention" "0.029 (-0.004 - 0.06)"         
##      Inpatient length of stay (days)
## [1,] "9.381 (9.254 - 9.535)"        
## [2,] "-0.009 (-0.013 - -0.005)"     
## [3,] "-0.156 (-0.259 - -0.055)"     
## [4,] "-0.013 (-0.019 - -0.006)"
```

``

## Table 2 - Risk adjusted model coefficients

```
caredays_mod_adj_ci <- confint(boot_list$caredays_adj,type = "perc")
readmit_mod_adj_ci <- confint(boot_list$readmit_adj,type = "perc")

cd_ci_adj <- bind_cols(coef = names(fixef(mod_list$caredays_adj)), 
                   caredays_mod_adj_ci[2:4]) %>%
  mutate(text = paste0(round(estimate,3)," (",round(lower,3)," - ",round(upper,3),")"))

re_ci_adj <- bind_cols(coef = names(fixef(mod_list$readmit_adj)), 
                   readmit_mod_adj_ci[2:4]) %>%
  mutate(text = paste0(round(estimate*100,3)," (",round(lower*100,3)," - ",round(upper*100,3),")"))

t2 <- cbind("Coefficient"=cd_ci_adj$coef,
      "30-day unplanned readmission (%)"=re_ci_adj$text,
      "Inpatient length of stay (days)"=cd_ci_adj$text)

t2
```

```
##       Coefficient              30-day unplanned readmission (%)
##  [1,] "(Intercept)"            "20.879 (19.23 - 22.543)"       
##  [2,] "since_start"            "-0.033 (-0.046 - -0.019)"      
##  [3,] "post_intervention"      "0.593 (0.143 - 1.043)"         
##  [4,] "since_intervention"     "0.028 (-0.006 - 0.058)"        
##  [5,] "age"                    "-0.094 (-0.114 - -0.073)"      
##  [6,] "gender"                 "-2.018 (-2.26 - -1.789)"       
##  [7,] "civil_statusDivorced"   "0.181 (-0.13 - 0.486)"         
##  [8,] "civil_statusUnmarried"  "-0.059 (-0.457 - 0.302)"       
##  [9,] "civil_statusWidow"      "-1.198 (-1.467 - -0.918)"      
## [10,] "planned_contact"        "-5.281 (-5.581 - -4.923)"      
## [11,] "n_diag"                 "1.152 (1.101 - 1.207)"         
## [12,] "n_op"                   "-0.229 (-0.288 - -0.168)"      
## [13,] "acsc_purdy"             "0.629 (0.203 - 1.056)"         
## [14,] "social_fctin_home"      "8.759 (8.436 - 9.091)"         
## [15,] "social_fctnursing_home" "3.218 (2.702 - 3.76)"          
##       Inpatient length of stay (days)
##  [1,] "5.445 (5.163 - 5.739)"        
##  [2,] "-0.021 (-0.026 - -0.016)"     
##  [3,] "-0.129 (-0.246 - -0.012)"     
##  [4,] "-0.009 (-0.017 - -0.002)"     
##  [5,] "-0.036 (-0.039 - -0.032)"     
##  [6,] "0.094 (0.061 - 0.127)"        
##  [7,] "-0.054 (-0.094 - -0.012)"     
##  [8,] "0.145 (0.08 - 0.21)"          
##  [9,] "-0.11 (-0.149 - -0.07)"       
## [10,] "-1.617 (-1.721 - -1.501)"     
## [11,] "0.591 (0.572 - 0.611)"        
## [12,] "1.344 (1.313 - 1.374)"        
## [13,] "-0.186 (-0.254 - -0.124)"     
## [14,] "2.207 (2.113 - 2.299)"        
## [15,] "4.954 (4.733 - 5.191)"
```

## Table 3 - Control group model coefficients

```
caredays_mod_soc_ci <- confint(boot_list$caredays_soc,type = "perc")
readmit_mod_soc_ci <- confint(boot_list$readmit_soc,type = "perc")

cd_ci_soc <- bind_cols(coef = names(fixef(mod_list$caredays_soc)), 
                   caredays_mod_soc_ci[2:4]) %>%
  mutate(text = paste0(round(estimate,3)," (",round(lower,3)," - ",round(upper,3),")"))

re_ci_soc <- bind_cols(coef = names(fixef(mod_list$readmit_soc)), 
                   readmit_mod_soc_ci[2:4]) %>%
  mutate(text = paste0(round(estimate*100,3)," (",round(lower*100,3)," - ",round(upper*100,3),")"))

t3 <- cbind("Coefficient"=cd_ci_soc$coef,
      "30-day unplanned readmission (%)"=re_ci_soc$text,
      "Inpatient length of stay (days)"=cd_ci_soc$text)

t3
```

```
##      Coefficient                     30-day unplanned readmission (%)
## [1,] "(Intercept)"                   "15.319 (14.896 - 15.743)"      
## [2,] "since_start"                   "-0.028 (-0.044 - -0.011)"      
## [3,] "post_intervention"             "0.322 (-0.248 - 0.884)"        
## [4,] "since_intervention"            "0.053 (0.013 - 0.089)"         
## [5,] "any_social"                    "7.828 (7.27 - 8.294)"          
## [6,] "since_start:any_social"        "0.011 (-0.015 - 0.037)"        
## [7,] "post_intervention:any_social"  "0.447 (-0.502 - 1.397)"        
## [8,] "since_intervention:any_social" "-0.036 (-0.094 - 0.017)"       
##      Inpatient length of stay (days)
## [1,] "7.578 (7.459 - 7.726)"        
## [2,] "-0.003 (-0.007 - 0)"          
## [3,] "-0.145 (-0.239 - -0.03)"      
## [4,] "0.007 (-0.001 - 0.012)"       
## [5,] "3.731 (3.489 - 3.913)"        
## [6,] "-0.014 (-0.02 - -0.006)"      
## [7,] "-0.018 (-0.26 - 0.155)"       
## [8,] "-0.033 (-0.044 - -0.019)"
```

# Additional analyses

## Causal graph

The covariate adjusted models are, admittedly, a bit of a causal
sallad. As noted in the manuscript, we do not recommend interpreting the
reported covariate coefficients as being causal, as their
interpretations depend on the ultimately unknown causal relationships
between them. We do however contend that all of the included covariates
are confounders with regards to both investigated outcomes in the
context of this longitudinal analysis. Consider the following simplified
causal graph:

Here, we consider the included covariates as either demographic
characteristics of the patient, or properties of the care episode. The
asymmetry between these groups being that demographics can affect the
care episode, but not vice-versa. While the internal relationships
between these groups of variables is difficult to characterize, we posit
that the direction of causality for each individual covariate flows from
covariate to the outcome in all cases. Given our interest in controlling
for covariate shifts over the pre- and post-CCA time frames in order to
obtain an unbiased estimate of the effect of the CCA reform, controlling
for all of these covariates ensures that no back-door paths between the
effect of the reform and post-reform outcomes exist among the observed
variables regardless of their internal relationships (i.e., even if
collider paths are opened, the terminal paths will always be mediating
or confounding).

Selecting a causal graph is, of course, more art than science. If you
consider a different DAG to be more appropriate, please contact the
corresponding author and we would be happy to estimate models using any
subset of these covariates for you.

## Population description

```
paste("Included admissions:",excl_n$orig)
```

```
## [1] "Included admissions: 2664583"
```

```
paste("Excluded due to missing hospital data:",excl_n$orig - excl_n$missingdata)
```

```
## [1] "Excluded due to missing hospital data: 616"
```

```
paste("Death before discharge:",excl_n$missingdata - excl_n$discharge)
```

```
## [1] "Death before discharge: 140035"
```

```
paste("Death before discharge percent:",(excl_n$missingdata - excl_n$discharge)/excl_n$orig)
```

```
## [1] "Death before discharge percent: 0.0525541895298439"
```

```
paste("Length of stay > 90 day:s",excl_n$discharge - excl_n$longstay)
```

```
## [1] "Length of stay > 90 day:s 2130"
```

```
paste("Observations during last 90 days of study period:",excl_n$longstay - excl_n$endofstudy)
```

```
## [1] "Observations during last 90 days of study period: 129537"
```

```
paste("Missing hospital ward data:",excl_n$endofstudy - excl_n$final)
```

```
## [1] "Missing hospital ward data: 6226"
```

```
paste("Final N:",excl_n$final)
```

```
## [1] "Final N: 2386039"
```

```
paste("Average admissions during July/august",mean(d_grp$n[!d_grp$month_dummy %in% c(7,8)]))
```

```
## [1] "Average admissions during July/august 28453.1063829787"
```

```
paste("Average during rest of year",mean(d_grp$n[d_grp$month_dummy %in% c(7,8)]))
```

```
## [1] "Average during rest of year 25105.1333333333"
```

```
paste("Average age",mean(d$age))
```

```
## [1] "Average age 78.4286103454302"
```

```
paste("Percent female at beginning of study",first(d_grp$gender))
```

```
## [1] "Percent female at beginning of study 0.511251951869202"
```

```
paste("Percent female at end of study",last(d_grp$gender))
```

```
## [1] "Percent female at end of study 0.550773694390716"
```

```
paste("Percent with social services",mean(d$any_social))
```

```
## [1] "Percent with social services 0.513982797431224"
```

```
paste("Aveage 30-day mortality rate",mean(d$mort30,na.rm = T))
```

```
## [1] "Aveage 30-day mortality rate 0.0440805871152986"
```

```
#R2 values (using the MuMIn package)

# Adjusted Readmission model
r.squaredGLMM(mod_list$readmit_adj)
```

```
## Warning: 'r.squaredGLMM' now calculates a revised statistic. See the help page.
```

```
##             R2m        R2c
## [1,] 0.02313825 0.04673969
```

```
# Adjusted length of stay model
r.squaredGLMM(mod_list$caredays_adj)
```

```
##            R2m       R2c
## [1,] 0.2518912 0.5505047
```

## Results noted in text

```
d_excess <- d %>%
  ungroup() %>%
  mutate(readmit_pred = predict(mod_list$readmit, newdata = .,type = "response"),
         caredays_pred = predict(mod_list$caredays, newdata = .,type = "response"),
         readmit_soc_pred = predict(mod_list$readmit_soc, newdata = .,type = "response"),
         caredays_soc_pred = predict(mod_list$caredays_soc, newdata = .,type = "response")) %>%
  # Set intervention effects to zero
  mutate(post_intervention = 0, 
         since_intervention = 0) %>%
  #Estimate counter-factual effects
  mutate(readmit_pred_cf = predict(mod_list$readmit, newdata = .,type = "response"),
         caredays_pred_cf = predict(mod_list$caredays, newdata = .,type = "response"),
         readmit_soc_pred_cf = predict(mod_list$readmit_soc, newdata = .,type = "response"),
         caredays_soc_pred_cf = predict(mod_list$caredays_soc, newdata = .,type = "response"))

saved_cd <- sum(d_excess$caredays_pred) - sum(d_excess$caredays_pred_cf) 
excess_readmits <- sum(d_excess$readmit_pred) - sum(d_excess$readmit_pred_cf)

mean_readmit_los <- mean(d_excess$next_admit_days[d_excess$unplanreadmit30 == 1 & 
                                                                d$post_intervention == 1])

mean_los_diff <- mean(d_excess$caredays_pred[d_excess$since_start == 36] - 
                      d_excess$caredays_pred_cf[d_excess$since_start == 36])*24

mean_readmit_diff <- mean(d_excess$readmit_pred[d_excess$since_start == 36] - 
                        d_excess$readmit_pred_cf[d_excess$since_start == 36])

mean_los_soc_diff <- mean(d_excess$caredays_soc_pred[d_excess$since_start == 36 & 
                                                     d_excess$any_social == 0]) -
                     mean(d_excess$caredays_soc_pred_cf[d_excess$since_start == 36 &
                                                          d_excess$any_social == 0])

paste("Excess care days:",saved_cd)
```

```
## [1] "Excess care days: -248521.030786924"
```

```
paste("Excess readmissions:",excess_readmits)
```

```
## [1] "Excess readmissions: 7572.34604351956"
```

```
paste("Average LoS for post_intervention readmissions:",mean_readmit_los)
```

```
## [1] "Average LoS for post_intervention readmissions: 7.73683403964376"
```

```
paste("Excess readmission care days:",excess_readmits*mean_readmit_los)
```

```
## [1] "Excess readmission care days: 58585.9846294639"
```

```
paste("LoS difference by end of study:",mean_los_diff)
```

```
## [1] "LoS difference by end of study: -3.73960611041227"
```

```
d_excess %>%
  group_by(since_start) %>%
  summarize(across(where(is.numeric),mean)) %>%
  pivot_longer(cols = c("readmit_pred","readmit_pred_cf","caredays_pred","caredays_pred_cf","caredays","unplanreadmit30"),
               names_to = "key",
               values_to = "value") %>%
  mutate(out = ifelse(key %in% c("readmit_pred","readmit_pred_cf","unplanreadmit30"),"Readmission","Length of stay")) %>%
  ggplot(aes(x=since_start,y=value,color = key)) +
  geom_line() +
  facet_wrap(~ out,scales = "free_y")
```

## Social care levels

```
# Note here significance may be inferred from t-values reported in the coefficient summaries below. Bootstrapped estimates tend to be a bit more conservative, but these effects are too strong to be at risk due to incorrect distributional assumptions.

summary(mod_list_sec$readmit_soc_fct, correlation=F)
```

```
## Linear mixed model fit by REML ['lmerMod']
## Formula: 
## unplanreadmit30 ~ (since_start + post_intervention + since_intervention) *  
##     social_fct + (1 | last_hosp_mvo) + (1 | month_dummy)
##    Data: d
## 
## REML criterion at convergence: 2256159
## 
## Scaled residuals: 
##     Min      1Q  Median      3Q     Max 
## -1.5854 -0.5718 -0.4203 -0.2546  2.5955 
## 
## Random effects:
##  Groups        Name        Variance   Std.Dev.
##  last_hosp_mvo (Intercept) 0.00426949 0.065341
##  month_dummy   (Intercept) 0.00002299 0.004795
##  Residual                  0.15053680 0.387991
## Number of obs: 2386039, groups:  last_hosp_mvo, 761; month_dummy, 12
## 
## Fixed effects:
##                                              Estimate  Std. Error t value
## (Intercept)                                0.15352899  0.00293608  52.290
## since_start                               -0.00027971  0.00004460  -6.272
## post_intervention                          0.00331958  0.00152738   2.173
## since_intervention                         0.00052298  0.00010944   4.779
## social_fctin_home                          0.08975639  0.00132538  67.721
## social_fctnursing_home                     0.03444965  0.00204734  16.827
## since_start:social_fctin_home              0.00006445  0.00006501   0.991
## since_start:social_fctnursing_home         0.00016886  0.00010073   1.676
## post_intervention:social_fctin_home        0.00210876  0.00218534   0.965
## post_intervention:social_fctnursing_home   0.01404875  0.00345135   4.071
## since_intervention:social_fctin_home      -0.00021847  0.00015831  -1.380
## since_intervention:social_fctnursing_home -0.00117807  0.00025581  -4.605
```

```
summary(mod_list_sec$caredays_soc_fct, correlation=F)
```

```
## Linear mixed model fit by REML ['lmerMod']
## Formula: caredays ~ (since_start + post_intervention + since_intervention) *  
##     social_fct + (1 | last_hosp_mvo) + (1 | month_dummy)
##    Data: d
## 
## REML criterion at convergence: 15956359
## 
## Scaled residuals: 
##     Min      1Q  Median      3Q     Max 
## -6.0226 -0.4986 -0.2002  0.2162 12.5996 
## 
## Random effects:
##  Groups        Name        Variance Std.Dev.
##  last_hosp_mvo (Intercept) 30.41904 5.5153  
##  month_dummy   (Intercept)  0.02172 0.1474  
##  Residual                  46.86715 6.8460  
## Number of obs: 2386039, groups:  last_hosp_mvo, 761; month_dummy, 12
## 
## Fixed effects:
##                                             Estimate Std. Error t value
## (Intercept)                                7.5572784  0.2052901  36.813
## since_start                               -0.0032141  0.0007876  -4.081
## post_intervention                         -0.1511457  0.0269695  -5.604
## since_intervention                         0.0074802  0.0019319   3.872
## social_fctin_home                          2.9581601  0.0233903 126.470
## social_fctnursing_home                     6.6748107  0.0361344 184.722
## since_start:social_fctin_home             -0.0104419  0.0011471  -9.103
## since_start:social_fctnursing_home        -0.0205169  0.0017775 -11.543
## post_intervention:social_fctin_home        0.0112762  0.0385611   0.292
## post_intervention:social_fctnursing_home  -0.1342630  0.0609004  -2.205
## since_intervention:social_fctin_home      -0.0259140  0.0027935  -9.277
## since_intervention:social_fctnursing_home -0.0502027  0.0045140 -11.122
```

## Undifferentiated readmissions

It seems suspicious that the proportion of planned admissions seen in
the descriptive statistics drops after 2018 - Could a shift towards
fewer admissions documented as planned account for the apparent increase
in unplanned readmissions? If so, we wouldn’t expect to see a change in
overall readmission rates:

```
    summary(mod_list_sec$readmit_all, correlation=F)
```

```
## Linear mixed model fit by REML ['lmerMod']
## Formula: readmit30 ~ since_start + post_intervention + since_intervention +  
##     (1 | last_hosp_mvo) + (1 | month_dummy)
##    Data: d
## 
## REML criterion at convergence: 2571826
## 
## Scaled residuals: 
##     Min      1Q  Median      3Q     Max 
## -1.6398 -0.5734 -0.5086 -0.2928  2.3316 
## 
## Random effects:
##  Groups        Name        Variance   Std.Dev.
##  last_hosp_mvo (Intercept) 0.00765624 0.087500
##  month_dummy   (Intercept) 0.00001973 0.004442
##  Residual                  0.17181422 0.414505
## Number of obs: 2386039, groups:  last_hosp_mvo, 761; month_dummy, 12
## 
## Fixed effects:
##                       Estimate  Std. Error t value
## (Intercept)         0.24091742  0.00354011  68.054
## since_start        -0.00025525  0.00003430  -7.442
## post_intervention   0.00353311  0.00116047   3.045
## since_intervention  0.00049065  0.00008152   6.019
```

```
    summary(mod_list_sec$readmit_all_soc, correlation=F)
```

```
## Linear mixed model fit by REML ['lmerMod']
## Formula: readmit30 ~ (since_start + post_intervention + since_intervention) *  
##     any_social + (1 | last_hosp_mvo) + (1 | month_dummy)
##    Data: d
## 
## REML criterion at convergence: 2551481
## 
## Scaled residuals: 
##     Min      1Q  Median      3Q     Max 
## -1.7263 -0.6138 -0.4579 -0.2279  2.4240 
## 
## Random effects:
##  Groups        Name        Variance   Std.Dev.
##  last_hosp_mvo (Intercept) 0.00773238 0.087934
##  month_dummy   (Intercept) 0.00001515 0.003893
##  Residual                  0.17035081 0.412736
## Number of obs: 2386039, groups:  last_hosp_mvo, 761; month_dummy, 12
## 
## Fixed effects:
##                                  Estimate  Std. Error t value
## (Intercept)                    0.20573911  0.00356117  57.773
## since_start                   -0.00042929  0.00004742  -9.052
## post_intervention              0.00238179  0.00162381   1.467
## since_intervention             0.00066617  0.00011641   5.722
## any_social                     0.07407530  0.00132216  56.026
## since_start:any_social         0.00025087  0.00006479   3.872
## post_intervention:any_social   0.00184652  0.00219022   0.843
## since_intervention:any_social -0.00024657  0.00015922  -1.549
```

```
    summary(mod_list_sec$readmit_all_adj, correlation=F)
```

```
## Linear mixed model fit by REML ['lmerMod']
## Formula: readmit30 ~ since_start + post_intervention + since_intervention +  
##     age + gender + civil_status + planned_contact + n_diag +  
##     n_op + acsc_purdy + social_fct + (1 | last_hosp_mvo) + (1 |  
##     month_dummy)
##    Data: d
## 
## REML criterion at convergence: 2529228
## 
## Scaled residuals: 
##     Min      1Q  Median      3Q     Max 
## -2.1220 -0.6021 -0.4555 -0.1996  2.5673 
## 
## Random effects:
##  Groups        Name        Variance   Std.Dev.
##  last_hosp_mvo (Intercept) 0.00743686 0.086237
##  month_dummy   (Intercept) 0.00001291 0.003593
##  Residual                  0.16883242 0.410892
## Number of obs: 2385139, groups:  last_hosp_mvo, 761; month_dummy, 12
## 
## Fixed effects:
##                           Estimate  Std. Error t value
## (Intercept)             0.33457155  0.00461299  72.528
## since_start            -0.00039790  0.00003399 -11.705
## post_intervention       0.00389614  0.00114933   3.390
## since_intervention      0.00048447  0.00008080   5.996
## age                    -0.00199117  0.00004060 -49.045
## gender                 -0.02496151  0.00057289 -43.571
## civil_statusDivorced   -0.00142132  0.00075287  -1.888
## civil_statusUnmarried  -0.00552288  0.00097052  -5.691
## civil_statusWidow      -0.01452744  0.00073456 -19.777
## planned_contact        -0.02536198  0.00081882 -30.974
## n_diag                  0.01185311  0.00011554 102.587
## n_op                   -0.00373659  0.00014128 -26.449
## acsc_purdy              0.00055843  0.00076755   0.728
## social_fctin_home       0.09417268  0.00065452 143.882
## social_fctnursing_home  0.05308985  0.00101057  52.535
```

```
## 
## Correlation matrix not shown by default, as p = 15 > 12.
## Use print(x, correlation=TRUE)  or
##     vcov(x)        if you need it
```

However, we see similar effects when investigating undifferentiated
readmission rates… So no smoking gun.

## Case-mix adjusted control group analysis

Case-mix adjustment could be considered to account for differences
between the control groups (as was done by for instance Ambugo et al.).
We can take a look at wether our results are sensitive to case mix
adjustment:

```
summary(mod_list_sec$caredays_soc_adj, correlation=F)
```

```
## Linear mixed model fit by REML ['lmerMod']
## Formula: caredays ~ (since_start + post_intervention + since_intervention) *  
##     any_social + age + gender + civil_status + planned_contact +  
##     n_diag + n_op + acsc_purdy + (1 | last_hosp_mvo) + (1 | month_dummy)
##    Data: d
## 
## REML criterion at convergence: 15352831
## 
## Scaled residuals: 
##     Min      1Q  Median      3Q     Max 
## -6.2068 -0.5315 -0.1391  0.3013 13.5700 
## 
## Random effects:
##  Groups        Name        Variance Std.Dev.
##  last_hosp_mvo (Intercept) 24.15961 4.9152  
##  month_dummy   (Intercept)  0.02099 0.1449  
##  Residual                  36.48047 6.0399  
## Number of obs: 2385139, groups:  last_hosp_mvo, 761; month_dummy, 12
## 
## Fixed effects:
##                                 Estimate Std. Error  t value
## (Intercept)                    4.6249329  0.1892850   24.434
## since_start                   -0.0148695  0.0006953  -21.387
## post_intervention             -0.1018576  0.0238056   -4.279
## since_intervention             0.0074306  0.0017050    4.358
## any_social                     3.1885354  0.0198548  160.593
## age                           -0.0283595  0.0005958  -47.602
## gender                         0.0708352  0.0084234    8.409
## civil_statusDivorced          -0.0301067  0.0110672   -2.720
## civil_statusUnmarried          0.2266125  0.0142613   15.890
## civil_statusWidow             -0.0836535  0.0107973   -7.748
## planned_contact               -1.6827592  0.0120548 -139.592
## n_diag                         0.6017094  0.0016990  354.160
## n_op                           1.3592799  0.0020798  653.578
## acsc_purdy                    -0.1973715  0.0112858  -17.488
## since_start:any_social        -0.0140831  0.0009485  -14.848
## post_intervention:any_social  -0.0326026  0.0320609   -1.017
## since_intervention:any_social -0.0342988  0.0023306  -14.717
```

```
## 
## Correlation matrix not shown by default, as p = 17 > 12.
## Use print(x, correlation=TRUE)  or
##     vcov(x)        if you need it
```

```
summary(mod_list_sec$readmit_soc_adj, correlation=F)
```

```
## Linear mixed model fit by REML ['lmerMod']
## Formula: 
## unplanreadmit30 ~ (since_start + post_intervention + since_intervention) *  
##     any_social + age + gender + civil_status + planned_contact +  
##     n_diag + n_op + acsc_purdy + (1 | last_hosp_mvo) + (1 | month_dummy)
##    Data: d
## 
## REML criterion at convergence: 2237591
## 
## Scaled residuals: 
##     Min      1Q  Median      3Q     Max 
## -1.6082 -0.5682 -0.4220 -0.2153  2.6896 
## 
## Random effects:
##  Groups        Name        Variance   Std.Dev.
##  last_hosp_mvo (Intercept) 0.00376939 0.061395
##  month_dummy   (Intercept) 0.00001542 0.003927
##  Residual                  0.14942389 0.386554
## Number of obs: 2385139, groups:  last_hosp_mvo, 761; month_dummy, 12
## 
## Fixed effects:
##                                  Estimate  Std. Error t value
## (Intercept)                    0.22110930  0.00396458  55.771
## since_start                   -0.00032622  0.00004444  -7.341
## post_intervention              0.00329701  0.00152170   2.167
## since_intervention             0.00048174  0.00010905   4.418
## any_social                     0.07672708  0.00127040  60.396
## age                           -0.00108677  0.00003811 -28.515
## gender                        -0.01972904  0.00053878 -36.618
## civil_statusDivorced           0.00135566  0.00070819   1.914
## civil_statusUnmarried         -0.00227650  0.00091258  -2.495
## civil_statusWidow             -0.01251868  0.00069098 -18.117
## planned_contact               -0.05142097  0.00076896 -66.871
## n_diag                         0.01131604  0.00010860 104.198
## n_op                          -0.00260236  0.00013261 -19.624
## acsc_purdy                     0.00650307  0.00072193   9.008
## since_start:any_social         0.00004053  0.00006070   0.668
## post_intervention:any_social   0.00471447  0.00205178   2.298
## since_intervention:any_social -0.00029841  0.00014914  -2.001
```

```
## 
## Correlation matrix not shown by default, as p = 17 > 12.
## Use print(x, correlation=TRUE)  or
##     vcov(x)        if you need it
```

It would appear not - Length of stay effects are, if anything,
slightly stronger than in the unadjusted model, while readmission
effects are slightly weaker.

## Secondary outcomes

```
summary(mod_list_sec$mortality, correlation=F)
```

```
## Linear mixed model fit by REML ['lmerMod']
## Formula: mort30 ~ since_start + post_intervention + since_intervention +  
##     (1 | last_hosp_mvo) + (1 | month_dummy)
##    Data: d
## 
## REML criterion at convergence: -824006.5
## 
## Scaled residuals: 
##     Min      1Q  Median      3Q     Max 
## -2.5294 -0.2622 -0.2076 -0.1321  4.9257 
## 
## Random effects:
##  Groups        Name        Variance    Std.Dev.
##  last_hosp_mvo (Intercept) 0.003913659 0.062559
##  month_dummy   (Intercept) 0.000007153 0.002675
##  Residual                  0.041387056 0.203438
## Number of obs: 2386039, groups:  last_hosp_mvo, 761; month_dummy, 12
## 
## Fixed effects:
##                       Estimate  Std. Error t value
## (Intercept)         0.04945660  0.00243812  20.285
## since_start         0.00002809  0.00001685   1.667
## post_intervention   0.00098560  0.00057005   1.729
## since_intervention -0.00006671  0.00004002  -1.667
```

```
summary(mod_list_sec$mortality_soc, correlation=F)
```

```
## Linear mixed model fit by REML ['lmerMod']
## Formula: mort30 ~ (since_start + post_intervention + since_intervention) *  
##     any_social + (1 | last_hosp_mvo) + (1 | month_dummy)
##    Data: d
## 
## REML criterion at convergence: -850544.7
## 
## Scaled residuals: 
##     Min      1Q  Median      3Q     Max 
## -2.5464 -0.3238 -0.1852 -0.0750  5.0708 
## 
## Random effects:
##  Groups        Name        Variance    Std.Dev.
##  last_hosp_mvo (Intercept) 0.003437752 0.058632
##  month_dummy   (Intercept) 0.000004944 0.002223
##  Residual                  0.040929837 0.202311
## Number of obs: 2386039, groups:  last_hosp_mvo, 761; month_dummy, 12
## 
## Fixed effects:
##                                  Estimate  Std. Error t value
## (Intercept)                    0.03064716  0.00228845  13.392
## since_start                   -0.00013800  0.00002326  -5.934
## post_intervention              0.00065960  0.00079628   0.828
## since_intervention             0.00025367  0.00005707   4.445
## any_social                     0.03954505  0.00064814  61.013
## since_start:any_social         0.00027422  0.00003176   8.634
## post_intervention:any_social   0.00040062  0.00107360   0.373
## since_intervention:any_social -0.00056694  0.00007805  -7.264
## optimizer (nloptwrap) convergence code: 0 (OK)
## Model failed to converge with max|grad| = 0.00200764 (tol = 0.002, component 1)
```

```
summary(mod_list_sec$mortality_adj, correlation=F)
```

```
## Linear mixed model fit by REML ['lmerMod']
## Formula: mort30 ~ since_start + post_intervention + since_intervention +  
##     age + gender + civil_status + planned_contact + n_diag +  
##     n_op + acsc_purdy + social_fct + (1 | last_hosp_mvo) + (1 |  
##     month_dummy)
##    Data: d
## 
## REML criterion at convergence: -898204.2
## 
## Scaled residuals: 
##     Min      1Q  Median      3Q     Max 
## -3.0287 -0.2902 -0.1644 -0.0527  5.2962 
## 
## Random effects:
##  Groups        Name        Variance    Std.Dev.
##  last_hosp_mvo (Intercept) 0.003433904 0.058600
##  month_dummy   (Intercept) 0.000004199 0.002049
##  Residual                  0.040112850 0.200282
## Number of obs: 2385139, groups:  last_hosp_mvo, 761; month_dummy, 12
## 
## Fixed effects:
##                            Estimate   Std. Error t value
## (Intercept)            -0.095689682  0.002710386 -35.305
## since_start            -0.000007996  0.000016585  -0.482
## post_intervention       0.000892746  0.000560767   1.592
## since_intervention     -0.000004075  0.000039401  -0.103
## age                     0.001573330  0.000019792  79.495
## gender                 -0.007890087  0.000279294 -28.250
## civil_statusDivorced   -0.004083094  0.000366988 -11.126
## civil_statusUnmarried  -0.000578838  0.000473083  -1.224
## civil_statusWidow      -0.007928539  0.000358053 -22.143
## planned_contact        -0.013714495  0.000399507 -34.329
## n_diag                  0.004199820  0.000056339  74.545
## n_op                   -0.000349623  0.000068937  -5.072
## acsc_purdy              0.004581899  0.000374183  12.245
## social_fctin_home       0.018263243  0.000319082  57.237
## social_fctnursing_home  0.095698787  0.000492681 194.241
```

```
## 
## Correlation matrix not shown by default, as p = 15 > 12.
## Use print(x, correlation=TRUE)  or
##     vcov(x)        if you need it
```

```
summary(mod_list_sec$ovplanned, correlation=F)
```

```
## Linear mixed model fit by REML ['lmerMod']
## Formula: ovplanned_30 ~ since_start + post_intervention + since_intervention +  
##     (1 | last_hosp_mvo) + (1 | month_dummy)
##    Data: d
## 
## REML criterion at convergence: 2760291
## 
## Scaled residuals: 
##     Min      1Q  Median      3Q     Max 
## -2.1599 -0.6334 -0.4731  1.1066  2.2182 
## 
## Random effects:
##  Groups        Name        Variance  Std.Dev.
##  last_hosp_mvo (Intercept) 0.0266555 0.16327 
##  month_dummy   (Intercept) 0.0002071 0.01439 
##  Residual                  0.1858663 0.43112 
## Number of obs: 2386039, groups:  last_hosp_mvo, 761; month_dummy, 12
## 
## Fixed effects:
##                       Estimate  Std. Error t value
## (Intercept)         0.32151685  0.00729540  44.071
## since_start         0.00051480  0.00003574  14.402
## post_intervention  -0.00499238  0.00120974  -4.127
## since_intervention -0.00003705  0.00008486  -0.437
```

```
summary(mod_list_sec$ovplanned_soc, correlation=F)
```

```
## Linear mixed model fit by REML ['lmerMod']
## Formula: 
## ovplanned_30 ~ (since_start + post_intervention + since_intervention) *  
##     any_social + (1 | last_hosp_mvo) + (1 | month_dummy)
##    Data: d
## 
## REML criterion at convergence: 2743902
## 
## Scaled residuals: 
##     Min      1Q  Median      3Q     Max 
## -2.2064 -0.6447 -0.4632  1.0954  2.3285 
## 
## Random effects:
##  Groups        Name        Variance  Std.Dev.
##  last_hosp_mvo (Intercept) 0.0251660 0.15864 
##  month_dummy   (Intercept) 0.0002014 0.01419 
##  Residual                  0.1845928 0.42964 
## Number of obs: 2386039, groups:  last_hosp_mvo, 761; month_dummy, 12
## 
## Fixed effects:
##                                  Estimate  Std. Error t value
## (Intercept)                    0.35861341  0.00715919  50.091
## since_start                    0.00053772  0.00004943  10.878
## post_intervention             -0.00513326  0.00169285  -3.032
## since_intervention            -0.00015701  0.00012125  -1.295
## any_social                    -0.07752826  0.00137649 -56.323
## since_start:any_social         0.00004554  0.00006745   0.675
## post_intervention:any_social   0.00035521  0.00227999   0.156
## since_intervention:any_social  0.00013944  0.00016575   0.841
```

```
summary(mod_list_sec$ovplanned_adj, correlation=F)
```

```
## Linear mixed model fit by REML ['lmerMod']
## Formula: ovplanned_30 ~ since_start + post_intervention + since_intervention +  
##     age + gender + civil_status + planned_contact + n_diag +  
##     n_op + acsc_purdy + social_fct + (1 | last_hosp_mvo) + (1 |  
##     month_dummy)
##    Data: d
## 
## REML criterion at convergence: 2703060
## 
## Scaled residuals: 
##     Min      1Q  Median      3Q     Max 
## -2.3947 -0.6677 -0.4488  1.0828  2.6401 
## 
## Random effects:
##  Groups        Name        Variance  Std.Dev.
##  last_hosp_mvo (Intercept) 0.0230759 0.15191 
##  month_dummy   (Intercept) 0.0001937 0.01392 
##  Residual                  0.1815349 0.42607 
## Number of obs: 2385139, groups:  last_hosp_mvo, 761; month_dummy, 12
## 
## Fixed effects:
##                           Estimate  Std. Error  t value
## (Intercept)             0.76999130  0.00759724  101.352
## since_start             0.00046521  0.00003535   13.160
## post_intervention      -0.00434655  0.00119585   -3.635
## since_intervention     -0.00011969  0.00008388   -1.427
## age                    -0.00593678  0.00004211 -140.997
## gender                  0.00025401  0.00059419    0.427
## civil_statusDivorced   -0.02544981  0.00078072  -32.598
## civil_statusUnmarried  -0.03980449  0.00100643  -39.550
## civil_statusWidow      -0.02295701  0.00076171  -30.139
## planned_contact         0.01033520  0.00085022   12.156
## n_diag                  0.00562786  0.00011987   46.950
## n_op                    0.00656848  0.00014671   44.771
## acsc_purdy             -0.02556693  0.00079607  -32.116
## social_fctin_home      -0.02342703  0.00067884  -34.510
## social_fctnursing_home -0.08971596  0.00104818  -85.592
```

```
## 
## Correlation matrix not shown by default, as p = 15 > 12.
## Use print(x, correlation=TRUE)  or
##     vcov(x)        if you need it
```

```
summary(mod_list_sec$ovunplanned, correlation=F)
```

```
## Linear mixed model fit by REML ['lmerMod']
## Formula: 
## ovunplanned_30 ~ since_start + post_intervention + since_intervention +  
##     (1 | last_hosp_mvo) + (1 | month_dummy)
##    Data: d
## 
## REML criterion at convergence: 2644604
## 
## Scaled residuals: 
##     Min      1Q  Median      3Q     Max 
## -1.6921 -0.6107 -0.5156 -0.2465  2.3011 
## 
## Random effects:
##  Groups        Name        Variance   Std.Dev.
##  last_hosp_mvo (Intercept) 0.00480041 0.069285
##  month_dummy   (Intercept) 0.00005193 0.007207
##  Residual                  0.17716144 0.420905
## Number of obs: 2386039, groups:  last_hosp_mvo, 761; month_dummy, 12
## 
## Fixed effects:
##                       Estimate  Std. Error t value
## (Intercept)         0.21859394  0.00338624  64.554
## since_start        -0.00008441  0.00003486  -2.422
## post_intervention   0.00269382  0.00118006   2.283
## since_intervention  0.00022994  0.00008280   2.777
```

```
summary(mod_list_sec$ovunplanned_soc, correlation=F)
```

```
## Linear mixed model fit by REML ['lmerMod']
## Formula: 
## ovunplanned_30 ~ (since_start + post_intervention + since_intervention) *  
##     any_social + (1 | last_hosp_mvo) + (1 | month_dummy)
##    Data: d
## 
## REML criterion at convergence: 2635970
## 
## Scaled residuals: 
##     Min      1Q  Median      3Q     Max 
## -1.6993 -0.6119 -0.5072 -0.2510  2.4033 
## 
## Random effects:
##  Groups        Name        Variance   Std.Dev.
##  last_hosp_mvo (Intercept) 0.00475713 0.068972
##  month_dummy   (Intercept) 0.00004731 0.006878
##  Residual                  0.17651788 0.420140
## Number of obs: 2386039, groups:  last_hosp_mvo, 761; month_dummy, 12
## 
## Fixed effects:
##                                  Estimate  Std. Error t value
## (Intercept)                    0.19545771  0.00338736  57.702
## since_start                   -0.00020347  0.00004831  -4.212
## post_intervention              0.00154622  0.00165459   0.935
## since_intervention             0.00036160  0.00011852   3.051
## any_social                     0.04877219  0.00134575  36.242
## since_start:any_social         0.00017286  0.00006595   2.621
## post_intervention:any_social   0.00198863  0.00222947   0.892
## since_intervention:any_social -0.00019356  0.00016207  -1.194
```

```
summary(mod_list_sec$ovunplanned_adj, correlation=F)
```

```
## Linear mixed model fit by REML ['lmerMod']
## Formula: 
## ovunplanned_30 ~ since_start + post_intervention + since_intervention +  
##     age + gender + civil_status + planned_contact + n_diag +  
##     n_op + acsc_purdy + social_fct + (1 | last_hosp_mvo) + (1 |  
##     month_dummy)
##    Data: d
## 
## REML criterion at convergence: 2615816
## 
## Scaled residuals: 
##     Min      1Q  Median      3Q     Max 
## -1.8646 -0.6209 -0.4929 -0.1986  2.4763 
## 
## Random effects:
##  Groups        Name        Variance   Std.Dev.
##  last_hosp_mvo (Intercept) 0.00386202 0.062145
##  month_dummy   (Intercept) 0.00003655 0.006046
##  Residual                  0.17510986 0.418461
## Number of obs: 2385139, groups:  last_hosp_mvo, 761; month_dummy, 12
## 
## Fixed effects:
##                           Estimate  Std. Error t value
## (Intercept)             0.28172163  0.00434828  64.789
## since_start            -0.00019392  0.00003467  -5.594
## post_intervention       0.00286409  0.00117301   2.442
## since_intervention      0.00020497  0.00008233   2.490
## age                    -0.00115143  0.00004134 -27.855
## gender                 -0.01491853  0.00058326 -25.578
## civil_statusDivorced    0.00513435  0.00076667   6.697
## civil_statusUnmarried  -0.00386577  0.00098832  -3.911
## civil_statusWidow      -0.01262930  0.00074807 -16.883
## planned_contact        -0.05865153  0.00083242 -70.459
## n_diag                  0.00942654  0.00011759  80.164
## n_op                   -0.00503394  0.00014358 -35.061
## acsc_purdy             -0.00719086  0.00078149  -9.202
## social_fctin_home       0.06266411  0.00066638  94.036
## social_fctnursing_home  0.00451940  0.00102880   4.393
```

```
## 
## Correlation matrix not shown by default, as p = 15 > 12.
## Use print(x, correlation=TRUE)  or
##     vcov(x)        if you need it
```

## Aggregate data analysis

```
# Check ability to detect effects in aggregate data per analysis plan. As a bonus, we can provide these data openly so at least this analysis can be easily reproduced!

# Here autocorrelation is an issue, so we'll use a sandwich estimator to adjust for non-iid observations. Could also build an appropriate time series model to account for autocorrelation, but we'll leave that to experts in time series modelling... If you are such an expert, please feel free to build better models and let us know what you see!

    caredays_grp <- lm(caredays ~ 
                         since_start + 
                         post_intervention + 
                         since_intervention +
                         month_dummy,
                       weights = n,
                         data = d_grp[d_grp$population == "All",])

    readmit_grp <- lm(unplanreadmit30 ~ 
                          since_start + 
                          post_intervention + 
                          since_intervention +
                         month_dummy,
                       weights = n,
                        data = d_grp[d_grp$population == "All",])
    
   caredays_soc_grp <- lm(caredays ~ 
                           (since_start + 
                            post_intervention + 
                            since_intervention) * any_social +
                         month_dummy,
                       weights = n,
                           data = d_grp[d_grp$population != "All",])

  readmit_soc_grp <- lm(unplanreadmit30 ~ 
                          (since_start + 
                            post_intervention + 
                            since_intervention) * any_social +
                         month_dummy,
                       weights = n,
                          data = d_grp[d_grp$population != "All",])
  
    caredays_adj_grp <- lm(caredays ~
                            since_start + 
                            post_intervention + 
                            since_intervention +  
                            age + 
                            gender + 
                            status_Unmarried +
                            status_Divorced +
                            planned_contact +
                            n_diag +
                            n_op +
                            acsc_purdy +
                            soc_in_home +
                            soc_nursing_home +
                         month_dummy,
                       weights = n,
                            data = d_grp[d_grp$population == "All",])
  
  readmit_adj_grp <- lm(unplanreadmit30 ~ 
                          since_start + 
                          post_intervention + 
                          since_intervention +  
                          age + 
                          gender + 
                          status_Unmarried +
                          status_Divorced +
                          planned_contact +
                          n_diag +
                          n_op +
                          acsc_purdy +
                          soc_in_home +
                          soc_nursing_home +
                         month_dummy,
                       weights = n,
                         data = d_grp[d_grp$population == "All",])
  
# The heteroskedasticity & Autocorrelation resistant (HAC) covariance matrix estimator seems like a good choice here
  
# Base length of stay model
round(coeftest(caredays_grp, vcov = vcovHAC(caredays_grp)),3)[1:4,] # no need to show month dummy CIs
```

```
##                    Estimate Std. Error t value Pr(>|t|)
## (Intercept)           8.062      0.038 209.807    0.000
## since_start          -0.004      0.002  -2.566    0.014
## post_intervention    -0.142      0.041  -3.443    0.001
## since_intervention   -0.017      0.003  -6.093    0.000
```

```
# Control group length of stay model  
round(coeftest(caredays_soc_grp, vcov = vcovHAC(caredays_soc_grp)),3)[c(1:4,17:19),]
```

```
##                               Estimate Std. Error t value Pr(>|t|)
## (Intercept)                      5.570      0.087  64.373    0.000
## since_start                     -0.001      0.002  -0.611    0.543
## post_intervention               -0.136      0.064  -2.117    0.037
## since_intervention               0.006      0.004   1.443    0.152
## since_start:any_social          -0.011      0.004  -2.781    0.007
## post_intervention:any_social    -0.033      0.118  -0.278    0.782
## since_intervention:any_social   -0.038      0.008  -4.987    0.000
```

```
# Case-mix adjusted length of stay model
round(coeftest(caredays_adj_grp, vcov = vcovHAC(caredays_adj_grp)),3)[c(1:14),]
```

```
##                    Estimate Std. Error t value Pr(>|t|)
## (Intercept)         -18.376     16.829  -1.092    0.283
## since_start          -0.017      0.005  -3.606    0.001
## post_intervention    -0.215      0.078  -2.777    0.009
## since_intervention   -0.006      0.008  -0.752    0.458
## age                   0.218      0.219   0.995    0.327
## gender                8.779      6.871   1.278    0.211
## status_Unmarried     19.839      7.730   2.567    0.015
## status_Divorced       4.807      6.510   0.738    0.466
## planned_contact      -7.391      2.617  -2.824    0.008
## n_diag               -0.055      0.852  -0.064    0.949
## n_op                  0.932      0.447   2.083    0.045
## acsc_purdy            2.609      2.103   1.241    0.224
## soc_in_home           2.379      1.664   1.430    0.163
## soc_nursing_home      4.584      7.309   0.627    0.535
```

```
# Base readmission model 
round(coeftest(readmit_grp, vcov = vcovHAC(readmit_grp)),3)[1:4,]
```

```
##                    Estimate Std. Error t value Pr(>|t|)
## (Intercept)           0.192      0.002 124.884    0.000
## since_start           0.000      0.000  -4.358    0.000
## post_intervention     0.006      0.001   4.777    0.000
## since_intervention    0.000      0.000   2.933    0.005
```

```
# Control group readmission model 
round(coeftest(readmit_soc_grp, vcov = vcovHAC(readmit_soc_grp)),3)[c(1:4,17:19),]
```

```
##                               Estimate Std. Error t value Pr(>|t|)
## (Intercept)                      0.152      0.001 126.366    0.000
## since_start                      0.000      0.000  -7.840    0.000
## post_intervention                0.003      0.002   2.249    0.027
## since_intervention               0.001      0.000   5.835    0.000
## since_start:any_social           0.000      0.000   2.148    0.034
## post_intervention:any_social     0.004      0.002   1.692    0.094
## since_intervention:any_social    0.000      0.000  -2.733    0.007
```

```
# Case-mix adjusted readmission model 
round(coeftest(readmit_adj_grp, vcov = vcovHAC(readmit_adj_grp)),3)[c(1:14),]
```

```
##                    Estimate Std. Error t value Pr(>|t|)
## (Intercept)          -0.355      0.815  -0.436    0.666
## since_start           0.000      0.000  -1.858    0.072
## post_intervention     0.006      0.002   3.045    0.005
## since_intervention    0.000      0.000   0.295    0.770
## age                   0.006      0.010   0.596    0.555
## gender                0.086      0.263   0.328    0.745
## status_Unmarried     -0.045      0.285  -0.158    0.876
## status_Divorced       0.350      0.198   1.769    0.086
## planned_contact      -0.071      0.101  -0.700    0.489
## n_diag                0.005      0.024   0.193    0.848
## n_op                 -0.017      0.014  -1.216    0.233
## acsc_purdy           -0.087      0.074  -1.173    0.250
## soc_in_home           0.062      0.077   0.807    0.426
## soc_nursing_home     -0.155      0.233  -0.667    0.510
```

While substantively similar, we see some differences between the
aggregate and individual level analyses: case mix adjusted analysis of
length of stay here appears to detect a level change in length of stay,
rather than a slope change. The slope reduction in the control group
analysis is here significant. These group level analysis are however
subject to additional forms of bias (eg simpsons paradox an other
ecological fallacies), for which reason we prefer to base our
conclusions on analyses of individual level data.

## Indivudual-level clustering

```
# To evaluate the sesitivity of the results to clustering at the individual level, we estimate model fixed coefficient CIs clustered at the individual level.

if(file.exists("final_ind_cluster_boot.Rda")){
  
  load("final_ind_cluster_boot.Rda")
    
}else{
  
  set.seed(42)
  
  ind_cluster_mod_list <- list()
    # Base models
    print(Sys.time())
    ind_cluster_mod_list$caredays <- lmer(caredays ~ 
                           since_start + 
                           post_intervention + 
                           since_intervention +
                         (1|lopnr) +
                         (1|month_dummy),
                         data = d)

    print(Sys.time())
    ind_cluster_mod_list$readmit <- lmer(unplanreadmit30 ~ 
                          since_start + 
                          post_intervention + 
                          since_intervention + 
                          (1|lopnr) +
                          (1|month_dummy), 
                        data = d)
  
  #Control series
  print(Sys.time())
  ind_cluster_mod_list$caredays_soc <- lmer(caredays ~ 
                           (since_start + 
                            post_intervention + 
                            since_intervention) * any_social +
                           (1|lopnr) +
                           (1|month_dummy),
                           data = d)
  
  print(Sys.time())
  ind_cluster_mod_list$readmit_soc <- lmer(unplanreadmit30 ~ 
                          (since_start + 
                             post_intervention + 
                             since_intervention) * any_social + 
                          (1|lopnr) +
                          (1|month_dummy),
                          data = d)
  
  # Risk adjusted
  print(Sys.time())
  ind_cluster_mod_list$caredays_adj <- lmer(caredays ~ 
                                since_start + 
                                post_intervention + 
                                since_intervention + 
                                age + 
                                gender + 
                                civil_status +
                                planned_contact +
                                n_diag +
                                n_op +
                                acsc_purdy +
                                social_fct +
                                (1|lopnr) +
                                (1|month_dummy),
                                data = d)
  
  print(Sys.time())
  ind_cluster_mod_list$readmit_adj <- lmer(unplanreadmit30 ~
                               since_start + 
                               post_intervention + 
                               since_intervention + 
                               age + 
                               gender + 
                               civil_status +
                               planned_contact +
                               n_diag +
                               n_op +
                               acsc_purdy +
                               social_fct +
                               (1|lopnr) +
                               (1|month_dummy),
                               data = d)
  print(Sys.time())
  
  #Setup cluster instances for foreach which, of course, uses a different paralellization scheme (doParallel) than multidbplyr.
  no_cores <- 7
  cl <- makeCluster(no_cores)
  
  registerDoParallel(cores = no_cores)
  
  # Run 143 bootstrap iterations on each core (1001 total). Note that this can take multiple days to run.
  
  boot_list_ind_cluster <- list()
  for (i in 1:length(ind_cluster_mod_list)){
    print(Sys.time())
    print(names(ind_cluster_mod_list[i]))
    boot_list_ind_cluster[names(ind_cluster_mod_list[i])] <- list(boot_parallel(ind_cluster_mod_list[[i]],cl))
    gc()
  }

  save(boot_list_ind_cluster,file="final_ind_cluster_boot.Rda")
  
}

caredays_mod_ci <- confint(boot_list_ind_cluster$caredays,type = "perc")
readmit_mod_ci <- confint(boot_list_ind_cluster$readmit,type = "perc")

cd_ci <- bind_cols(coef = names(fixef(mod_list$caredays)), 
                   caredays_mod_ci[2:4]) %>%
  mutate(text = paste0(round(estimate,3)," (",round(lower,3)," - ",round(upper,3),")"))

re_ci <- bind_cols(coef = names(fixef(mod_list$readmit)), 
                   readmit_mod_ci[2:4]) %>%
  mutate(text = paste0(round(estimate*100,3)," (",round(lower*100,3)," - ",round(upper*100,3),")"))

t1_ind <- cbind("Coefficient"=cd_ci$coef,
      "30-day unplanned readmission (%)"=re_ci$text,
      "Inpatient length of stay (days)"=cd_ci$text)

t1_ind
```

```
##      Coefficient          30-day unplanned readmission (%)
## [1,] "(Intercept)"        "16.601 (11.803 - 13.147)"      
## [2,] "since_start"        "-0.006 (0.047 - 0.099)"        
## [3,] "post_intervention"  "0.589 (-0.021 - 1.013)"        
## [4,] "since_intervention" "0.049 (-0.023 - 0.078)"        
##      Inpatient length of stay (days)
## [1,] "7.6 (7.043 - 7.266)"          
## [2,] "-0.002 (0.01 - 0.019)"        
## [3,] "-0.143 (-0.286 - -0.04)"      
## [4,] "-0.015 (-0.029 - -0.011)"
```

```
## Table 2 - Risk adjusted model coefficients

caredays_mod_adj_ci <- confint(boot_list_ind_cluster$caredays_adj,type = "perc")
readmit_mod_adj_ci <- confint(boot_list_ind_cluster$readmit_adj,type = "perc")

cd_ci_adj <- bind_cols(coef = names(fixef(mod_list$caredays_adj)), 
                   caredays_mod_adj_ci[2:4]) %>%
  mutate(text = paste0(round(estimate,3)," (",round(lower,3)," - ",round(upper,3),")"))

re_ci_adj <- bind_cols(coef = names(fixef(mod_list$readmit_adj)), 
                   readmit_mod_adj_ci[2:4]) %>%
  mutate(text = paste0(round(estimate*100,3)," (",round(lower*100,3)," - ",round(upper*100,3),")"))

t2_ind <- cbind("Coefficient"=cd_ci_adj$coef,
      "30-day unplanned readmission (%)"=re_ci_adj$text,
      "Inpatient length of stay (days)"=cd_ci_adj$text)

t2_ind
```

```
##       Coefficient              30-day unplanned readmission (%)
##  [1,] "(Intercept)"            "15.179 (-1.645 - 3.546)"       
##  [2,] "since_start"            "-0.025 (0.017 - 0.067)"        
##  [3,] "post_intervention"      "0.587 (0.025 - 1.103)"         
##  [4,] "since_intervention"     "0.045 (-0.03 - 0.076)"         
##  [5,] "age"                    "-0.041 (0.073 - 0.127)"        
##  [6,] "gender"                 "-2.133 (-2.37 - -2.009)"       
##  [7,] "civil_statusDivorced"   "0.318 (0.438 - 0.931)"         
##  [8,] "civil_statusUnmarried"  "0.234 (0.502 - 1.135)"         
##  [9,] "civil_statusWidow"      "-1.13 (-0.974 - -0.429)"       
## [10,] "planned_contact"        "-4.347 (-0.3 - 3.172)"         
## [11,] "n_diag"                 "0.918 (0.412 - 0.596)"         
## [12,] "n_op"                   "-0.097 (0.12 - 0.353)"         
## [13,] "acsc_purdy"             "0.635 (-0.01 - 0.864)"         
## [14,] "social_fctin_home"      "7.831 (6.191 - 6.993)"         
## [15,] "social_fctnursing_home" "2.05 (-0.654 - 0.718)"         
##       Inpatient length of stay (days)
##  [1,] "4.489 (3.553 - 4.172)"        
##  [2,] "-0.021 (-0.029 - -0.021)"     
##  [3,] "-0.128 (-0.227 - 0.011)"      
##  [4,] "-0.01 (-0.021 - -0.007)"      
##  [5,] "-0.047 (-0.046 - -0.037)"     
##  [6,] "0.108 (0.103 - 0.165)"        
##  [7,] "0.046 (0.028 - 0.13)"         
##  [8,] "0.306 (0.32 - 0.443)"         
##  [9,] "-0.123 (-0.132 - -0.018)"     
## [10,] "-1.72 (-1.757 - -1.581)"      
## [11,] "0.652 (0.692 - 0.729)"        
## [12,] "1.219 (1.285 - 1.325)"        
## [13,] "-0.398 (-0.332 - -0.205)"     
## [14,] "2.91 (2.519 - 2.747)"         
## [15,] "5.945 (5.177 - 5.67)"
```

```
caredays_mod_soc_ci <- confint(boot_list_ind_cluster$caredays_soc,type = "perc")
readmit_mod_soc_ci <- confint(boot_list_ind_cluster$readmit_soc,type = "perc")

cd_ci_soc <- bind_cols(coef = names(fixef(mod_list$caredays_soc)), 
                   caredays_mod_soc_ci[2:4]) %>%
  mutate(text = paste0(round(estimate,3)," (",round(lower,3)," - ",round(upper,3),")"))

re_ci_soc <- bind_cols(coef = names(fixef(mod_list$readmit_soc)), 
                   readmit_mod_soc_ci[2:4]) %>%
  mutate(text = paste0(round(estimate*100,3)," (",round(lower*100,3)," - ",round(upper*100,3),")"))

t3_ind <- cbind("Coefficient"=cd_ci_soc$coef,
      "30-day unplanned readmission (%)"=re_ci_soc$text,
      "Inpatient length of stay (days)"=cd_ci_soc$text)

t3_ind
```

```
##      Coefficient                     30-day unplanned readmission (%)
## [1,] "(Intercept)"                   "13.541 (10.182 - 11.328)"      
## [2,] "since_start"                   "-0.021 (0.01 - 0.064)"         
## [3,] "post_intervention"             "0.276 (-0.692 - 0.858)"        
## [4,] "since_intervention"            "0.075 (0.001 - 0.118)"         
## [5,] "any_social"                    "6.898 (4.266 - 5.745)"         
## [6,] "since_start:any_social"        "0.012 (-0.006 - 0.063)"        
## [7,] "post_intervention:any_social"  "0.547 (-0.487 - 1.957)"        
## [8,] "since_intervention:any_social" "-0.044 (-0.138 - 0.012)"       
##      Inpatient length of stay (days)
## [1,] "5.336 (5.281 - 5.513)"        
## [2,] "-0.002 (-0.005 - 0.005)"      
## [3,] "-0.132 (-0.261 - 0.006)"      
## [4,] "0.009 (0.003 - 0.022)"        
## [5,] "4.949 (4.729 - 5.125)"        
## [6,] "-0.012 (-0.024 - -0.01)"      
## [7,] "-0.041 (-0.256 - 0.229)"      
## [8,] "-0.041 (-0.061 - -0.026)"
```
