# Supplementary material for: The Impact of the Swedish Care Coordination Act on Hospital Readmission and Length-of-Stay among Multi-Morbid Elderly Patients: A Controlled Interrupted Time Series Analysis
Source: Int J Integr Care. 2023 May 23;23(2):17. doi: 10.5334/ijic.6510 (PMC10216000; doi:10.5334/ijic.6510)
Supplement: Appendix 1. — Analysis notebook. [file ijic-23-2-6510-s1.zip › AppendixFiles/Appendix_files/index.html]

Ginger Extension Writer


Back to Website

Go Premium

Synonyms

Dictionary

Translate


Select a word or expression or  
 Type directly in the search bar.

*0/600*
 Your text was partially checked.
 Characters

Rephrase
